# Four-Objective Optimizations for an Improved Irreversible Closed Modified Simple Brayton Cycle

**DOI:** 10.3390/e23030282

**Published:** 2021-02-26

**Authors:** Chenqi Tang, Lingen Chen, Huijun Feng, Yanlin Ge

**Affiliations:** 1 Institute of Thermal Science and Power Engineering, Wuhan Institute of Technology, Wuhan 430205, China; tangchenqi7@163.com (C.T.); geyali9@hotmail.com (Y.G.); 2 School of Mechanical & Electrical Engineering, Wuhan Institute of Technology, Wuhan 430205, China; 3 College of Power Engineering, Naval University of Engineering, Wuhan 430033, China

**Keywords:** closed simple Brayton cycle, power output, thermal efficiency, power density, ecological function, multi-objective optimization

## Abstract

An improved irreversible closed modified simple Brayton cycle model with one isothermal heating process is established in this paper by using finite time thermodynamics. The heat reservoirs are variable-temperature ones. The irreversible losses in the compressor, turbine, and heat exchangers are considered. Firstly, the cycle performance is optimized by taking four performance indicators, including the dimensionless power output, thermal efficiency, dimensionless power density, and dimensionless ecological function, as the optimization objectives. The impacts of the irreversible losses on the optimization results are analyzed. The results indicate that four objective functions increase as the compressor and turbine efficiencies increase. The influences of the latter efficiency on the cycle performances are more significant than those of the former efficiency. Then, the NSGA-II algorithm is applied for multi-objective optimization, and three different decision methods are used to select the optimal solution from the Pareto frontier. The results show that the dimensionless power density and dimensionless ecological function compromise dimensionless power output and thermal efficiency. The corresponding deviation index of the Shannon Entropy method is equal to the corresponding deviation index of the maximum ecological function.

## 1. Introduction

Some scholars have studied performances of gas turbine plants (Brayton cycle (BCY)) [1,2,3,4] all over the world for their small size and comprehensive energy sources. The gas-steam combined, cogeneration, and other complex cycles have appeared for the requirements of energy conservation and environmental protection. The thermal efficiency (η) of a simple BCY is low, and the NOx content in combustion product is high. To further improve the cycle performance, it has become a key research direction to improve the initial temperature of the gas or to adopt the advanced cycles (such as regenerative, intercooled, intercooled and regenerative, isothermal heating, and other complex combined cycles).

In the case of simple heating, when the compressible subsonic gas flows through the smooth heating pipe with the fixed cross-sectional area, the gas temperature increases along the pipe direction; in the case of simple region change, when the compressible subsonic gas flows through the smooth adiabatic reductive pipe, the gas temperature decreases along the pipe direction. Based on these two gas properties, the isothermal heating process (IHP) can be realized when the compressible subsonic gas flows through the smooth heating reductive pipe. The combustion chamber, which can recognize the IHP, is called the convergent combustion chamber (CCC). The pipe of the CCC is assumed to be smooth. During the heating process, the temperature of the gas is always constant. According to the energy conservation law, the kinetic energy of the gas increases, that is, the pushing work of the gas increases. From the definition of enthalpy, it can be seen that enthalpy includes two parts: the thermodynamic energy and the pushing work. Therefore, the enthalpy increases. Based on this, Vecchiarelli et al. [5] proposed the CCC to perform the IHP of the working fluid. The power output ($\bar{W}$) and η of the BCY could be improved, and the emission of harmful gases such as NOx could be reduced by adding this combustion chamber model. The regenerative BCYs [6,7,8] and binary BCY [9] with IHPs were also studied by applying the classical thermodynamics.

Finite time thermodynamics (FTT) is a useful thermodynamic analysis theory and method [10,11,12,13,14,15,16,17,18,19]. In general, it is known that Curzon and Ahlborn [12] initialized FTT in 1975. In fact, the classical efficiency bound at the maximum power was also derived by Moutier [10] in 1872 and Novikov [11] in 1957. The applications of FTT include majorly two fields: optimal configurations [20,21,22,23,24,25,26,27,28,29,30,31,32,33,34,35,36] and optimal performances [37,38,39,40,41,42,43,44,45,46,47,48,49,50,51,52,53,54,55,56,57,58,59,60,61] studies for thermodynamic cycles and processes. The W and η have been often considered as the optimization objectives (OPOs) of the heat engines [62,63,64,65,66,67,68,69,70,71,72]. When the power density (P) [73,74,75,76,77,78,79,80,81] was taken as the OPO, the operating unit had a smaller size and higher η. Aditionally, the ecological function (E) [82,83,84,85,86,87,88] is also an OPO that balances the conflict between W and η.

Kaushik et al. [89] first applied the FTT to studying the regenerative BCY with an IHP. The regenerative, intercooled and regenerative complex BCYs with isothermal heating combustor were further investigated [90,91,92,93,94,95,96]. Based on this, Chen et al. [97,98,99] studied the endoreversible simple isothermal heating BCY with the W, η and E as OPOs. Arora et al. [100,101] adopted NSGA-II and evolutionary algorithms to optimize the irreversible isothermal heating regenerative BCY with the W and η as the OPOs. Chen et al. [102] considered the variable isothermal pressure drop ratio ($\pi_{t}$), established an improved isothermal heating regenerative BCY model, and studied the regenerator’s role on cycle performance. Qi et al. [103] demonstrated a closed endoreversible modified binary BCY with IHPs and found the W and η raised as the heat reservoirs’ temperature ratios. Tang et al. [104] considered the variable $\pi_{t}$ and established an improved irreversible binary BCY model modified by isothermal heating. The heat exchanger’s heat conductance distributions (HCDs) and the top and bottom cycles’ pressure ratios were taken as optimization variables to optimize the cycle performance.

In the process of the thermodynamic system optimization, single-objective optimization often led to unacceptable objectives for other objectives when there were conflicts among the considered goals. Multi-objective optimization would consider the trade-offs among the goals, and the optimized results were more reasonable [99,100,102,105,106,107,108,109,110,111,112,113,114,115,116,117,118,119,120,121,122,123,124,125].

In applying the FTT, the heat transfer was introduced into the thermodynamic analysis of the thermodynamic process, and finite temperature difference was considered in Refs. [11,12]. In this paper, the same method in Refs. [11,12] will be used, and the finite temperature difference will be considered when establishing the model, which is the key relation among this paper and the Refs. [11,12]. On this basis, the cycle’s irreversibility will be further considered, and the corresponding conclusion will be more in line with the actual situation. The compression and expansion losses in the model in Refs. [97,98,99] were not considered, and they will be further considered in this paper alongside the losses in the heat exchangers. Meanwhile, the thermal resistance loss and the optimal HCD will be considered. With the W, η, P and E, respectively, as the OPOs, an improved irreversible closed modified simple BCY with one IHP and coupled to variable-temperature heat reservoirs (VTHRs) will be optimized, and the optimization results will be compared. The effects of the compressor and turbine efficiencies on optimization results will be analyzed. The NSGA-II algorithm will be applied for multi-objective optimization to obtain the Pareto frontier further. The results obtained in this paper will reveal the original results in Refs. [10,11,12], which were the initial work of the FTT theory.

## 2. Cycle Model and Performance Analytical Indicators

**Figure 1:**
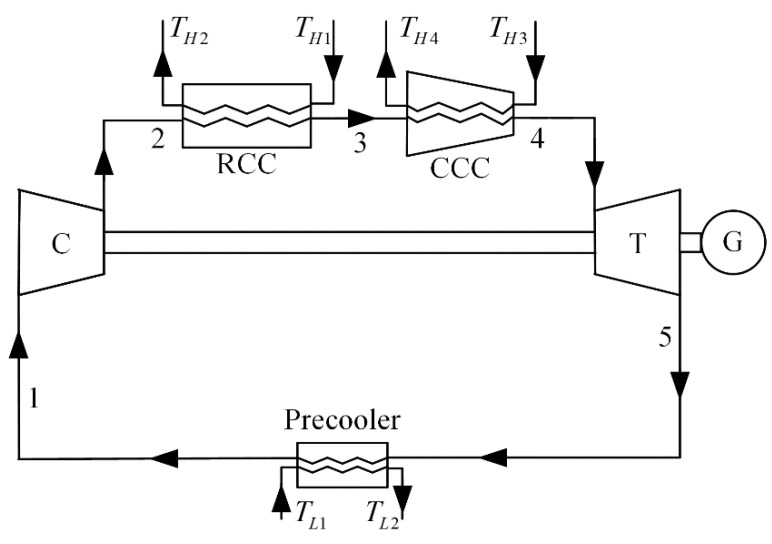
Schematic diagram of the cycle.

**Figure 2:**
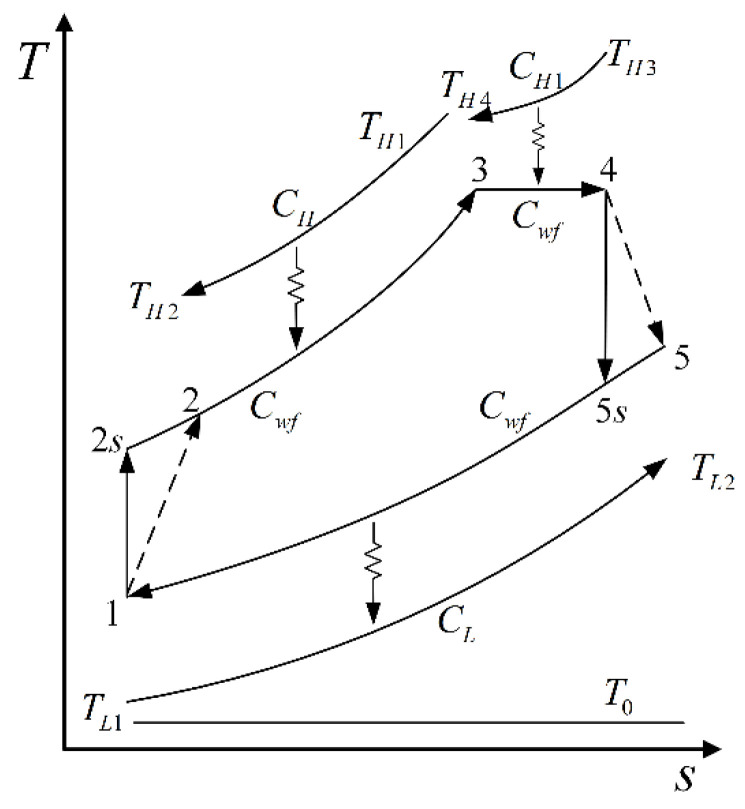
Diagram of the cycle.

The schematic diagram of an improved irreversible closed modified simple BCY with one IHP and coupled to VTHRs is shown in Figure 1. A compressor (C), a regular combustion chamber (RCC), a CCC, a turbine (T), and a precooler are the main parts of the cycle. The corresponding T−s diagram of the cycle is shown in Figure 2. The cycle consists of five processes in total:

1. The process 1→2 is an irreversible adiabatic compression process in C, and the process 1→2s is an isentropic process corresponding to the process 1→2.
2. The process 2→3 is an isobaric endothermic process in RCC.
3. The process 3→4 is an IHP in CCC. In CCC, the working fluid is isothermally heated, and its flow velocity rises from $V_{3}$ to $V_{4}$ (the Mach number increases from $M_{3}$ to $M_{4}$), and its specific enthalpy rises from $h_{3}$ to $h_{4}$. The parameter $\pi_{t}(=p_{4}/p_{3}\leq 1)$ is the isothermal pressure drop ratio. The $\pi_{t}$ needs to be given in Refs. [97,98], but the $\pi_{t}$ of the improved cycle established in this paper will change with the operation state. The degree of the IHP can be represented by $\pi_{t}$, and the greater the $\pi_{t}$, the greater the degree.
4. The process 4→5 is an adiabatic exothermic process in turbine, and the process 4→5s is the isentropic process corresponding to the process 4→5.
5. The process 5→1 is an isobaric exothermic process in a precooler.

The working fluid is the ideal gas. The pressures and temperatures of the working fluid are $p_{i}\left(i=1,\;2,\;3,\;4,\;5,\;2s,\;5s\right)$ and $T_{i}$, and the ratio of specific heat is k. The outside fluids’ temperatures are $T_{j}(j=H1,\;H2,\;H3,\;H4,\;L1,\;L2)$. The specific heat at constant pressure and the working fluid’s mass flow rate are $C_{p}$ and $\dot{m}$. The working fluid’s thermal capacity rate is $C_{wf}$ where $C_{wf}=C_{p}\dot{m}$. The outer fluids’ thermal capacity rates at the RCC, CCC, and precooler are $C_{H}$, $C_{H1}$ and $C_{L}$, respectively; then, one has:

(1) $$C_{H\max}=\max\{C_{H},\;C_{wf}\},\;C_{L\max}=\max\{C_{L},\;C_{wf}\},\;C_{H\min}=\min\{C_{H},\;C_{wf}\},\;C_{L\min}=\min\{C_{L},\;C_{wf}\}$$

The heat exchangers’ heat conductance is the product of the heat transfer coefficient and the heat transfer area. The heat exchangers’ heat conductance values in the RCC, CCC, and precooler are $U_{H}$, $U_{H1}$ and $U_{L}$, the heat transfer units’ numbers are $N_{H}$, $N_{H1}$ and $N_{L}$, and the effectiveness values are $E_{H}$, $E_{H1}$ and $E_{L}$, respectively:

(2) $$N_{H}=U_{H}/C_{H\min},\;N_{H1}=U_{H1}/C_{H1},\;N_{L}=U_{L}/C_{L\min}$$

(3) $$E_{H}=\frac{1-e^{-N_{H}(1-C_{H\min}/C_{H\max})}}{1-(C_{H\min}/C_{H\max})e^{-N_{H}(1-C_{H\min}/C_{H\max})}}$$

(4) $$E_{H1}=1-e^{-N_{H1}}$$

(5) $$E_{L}=\frac{1-e^{-N_{L}(1-C_{L\min}/C_{L\max})}}{1-(C_{L\min}/C_{L\max})e^{-N_{L}(1-C_{L\min}/C_{L\max})}}$$

When $C_{H\max}=C_{H\min}$ and $C_{L\max}=C_{L\min}$, Equations (3) and (5) are, respectively, simplified as:

(6) $$E_{H}=N_{H}/(N_{H}+1)$$

(7) $$E_{L}=N_{L}/(N_{L}+1)$$

The outside fluids’ temperature ratios at the RCC and CCC are:

(8) $$\tau_{H1}=T_{H1}/T_{0}$$

(9) $$\tau_{H3}=T_{H3}/T_{0}$$

where $T_{0}$ is the ambient temperature.

The process 1→2s is the isentropic one, namely:

(10) $$T_{2s}/T_{1}=\pi^{m}=x$$

where m=(k−1)/k and π is the pressure ratio of the compressor.

The process 4→5s is the isentropic one, namely:

(11) $$T_{4}/T_{5s}=\pi^{m}\pi_{t}^{m}=xy$$

The process 3→4 is the isothermal one, namely:

(12) $$T_{3}=T_{4}$$

(13) $$\;{\dot{Q}}_{3-4}=\dot{m}(h_{4}-h_{3})-\dot{m}\int_{3}^{4}vdp=-\dot{m}R_{g}T_{3}\ln\pi_{t}$$

where $\pi_{t}$, $M_{3}$ and $M_{4}$ must satisfy the following relation:

(14) $$\ln\pi_{t}=-c_{p}(k-1)(M_{4}^{2}-M_{3}^{2})/(2R_{g})$$

where the working fluid’s flow velocity must be subsonic, namely, $M_{3},\;M_{4}<1$ Because the working fluid has an initial speed, $(M_{4}^{2}-M_{3}^{2})<0.96$ and $\pi_{t}>0.5107$ when $M_{3}=0.2$. Because of $M_{4}>M_{3}$, $\pi_{t}<1$. When $\pi_{t}=1$, the cycle model in this paper can be simplified to a simple Brayton cycle.

According to the definition of $\pi_{t}$, it can be obtained that:

(15) $$\pi_{t}=\frac{p_{4}}{p_{3}}=\frac{p_{4}}{p_{3}}\cdot \frac{p_{1}}{p_{1}}=\frac{p_{4}}{p_{1}}\cdot \pi^{-1}\geq \pi^{-1}$$

Considering the irreversibilities in the compressor and the turbine, the efficiencies of them are:

(16) $$\eta_{c}=(T_{1}-T_{2s})/(T_{1}-T_{2})$$

(17) $$\eta_{t}=(T_{5}-T_{4})/(T_{5s}-T_{4})$$

The pressure drop is not considered in this paper. It will be considered in future, as it was by Ref. [126]. The study in Ref. [126] showed that the pressure drop loss has a little influence on the cycle performance quantitatively, and has no influence qualitatively. 

The working fluid’s heat absorption rates at RCC and CCC are ${\dot{Q}}_{2-3}$ and ${\dot{Q}}_{3-4}$, respectively:

(18) $${\dot{Q}}_{2-3}=C_{H}(T_{H1}-T_{H2})=C_{wf}(T_{3}-T_{2})=C_{H\min}E_{H}(T_{H1}-T_{2})$$

(19) $${\dot{Q}}_{3-4}=C_{H1}(T_{H3}-T_{H4})=C_{H1}E_{H1}(T_{H3}-T_{3})=\dot{m}(V_{{}^{4}}^{2}-V_{3}^{2})/2$$

The heat releasing rate at the precooler is ${\dot{Q}}_{5-1}$, namely:

(20) $${\dot{Q}}_{5-1}=C_{L}(T_{L2}-T_{L1})=C_{wf}(T_{5}-T_{1})=C_{L\min}E_{L}(T_{5}-T_{L1})$$

The heat leakages between the heat source and the environment [127,128] are neglected. Therefore, the W and η are:

(21) $$W={\dot{Q}}_{2-3}+{\dot{Q}}_{3-4}-{\dot{Q}}_{5-1}$$

(22) $$\eta =W/({\dot{Q}}_{2-3}+{\dot{Q}}_{3-4})$$

The dimensionless power output ($\bar{W}$) is:

(23) $$\bar{W}=W/(C_{wf}T_{0})$$

The maximum specific volume corresponding to state point 5 is $v_{5}$. The P is calculated as:

(24) $$P=W/v_{5}$$

The specific volume corresponding to state point 1 is $v_{1}$. The dimensionless power density ($\bar{P}$) and dimensionless maximum specific volume ($v_{5}/v_{1}$) are obtained as:

(25) $$\bar{P}=\frac{P}{C_{wf}T_{0}/v_{1}}=\frac{W/v_{5}}{C_{wf}T_{0}/v_{1}}=\frac{W}{C_{wf}T_{0}}\times \frac{T_{1}}{T_{5}}=\bar{W}\times \frac{T_{1}}{T_{5}}$$

(26) $$v_{5}/v_{1}=T_{5}/T_{1}$$

There are two different methods for calculating the entropy production rate. One was suggested by Bejan [129,130], and the another was suggested by Salamon et al. [131]. In this article, the method used is the one suggested by the latter.

The entropy production rate ($s_{g}$) and E are, respectively, calculated as:

(27) $$s_{g}=C_{H}\ln(T_{H2}/T_{H1})+C_{H1}\ln(T_{H4}/T_{H3})+C_{L}\ln(T_{L2}/T_{L1})$$

(28) $$E=W-T_{0}s_{g}$$

The dimensionless ecological function ($\bar{E}$) is obtained as:

(29) $$\bar{E}=E/(C_{wf}T_{0})$$

Equations (10)–(12) and (16)–(29) are combined, and the four dimensionless performance indicators of the cycle are obtained as follows:

(30) $$\bar{W}=\frac{\begin{array}{l} C_{wf}xy(C_{H1}E_{H1}T_{H3}+C_{L\min}E_{L}T_{L1})+C_{H\min}E_{H}T_{H1}\{xy[C_{wf}\\ -C_{H1}E_{H1}+C_{L\min}E_{L}(\eta_{t}-1)]-C_{L\min}E_{L}\eta_{t}\}+a_{1}\{C_{L\min}E_{L}\\ \times [(\eta_{t}-1)xy-\eta_{t}](C_{wf}-E_{H}C_{H\min})-xy[C_{wf}C_{H\min}E_{H}\\ +C_{H1}E_{H1}(C_{wf}-C_{H\min}E_{H})]\} \end{array}}{C_{wf}^{2}T_{0}xy}$$

(31) $$\eta =\frac{\begin{array}{l} C_{H\min}C_{L\min}E_{H}E_{L}\eta_{t}T_{H1}-\{C_{H\min}E_{H}T_{H1}[C_{wf}-C_{H1}E_{H1}+C_{L\min}E_{L}(\eta_{t}-1)]\\ \begin{array}{l} +C_{wf}xy(C_{H1}E_{H1}T_{H3}+C_{L\min}E_{L}T_{L1})\}+a_{1}\{[C_{H\min}C_{wf}E_{H}+C_{H1}E_{H1}(C_{wf}\\ -E_{H}C_{H\min})]xy-C_{L\min}E_{L}(C_{wf}-C_{H\min}E_{H})[(\eta_{t}-1)xy-\eta_{t}]\} \end{array} \end{array}}{\begin{array}{l} xy\{a_{1}[C_{H1}C_{wf}E_{H1}+C_{H\min}E_{H}(C_{wf}-C_{H1}E_{H1})]+C_{H\min}E_{H}(C_{H1}E_{H1}\\ -C_{wf})T_{H1}-C_{H1}C_{wf}E_{H1}T_{H3}\} \end{array}}$$

(32) $$\bar{P}=\frac{\begin{array}{l} \{a_{1}(C_{wf}-C_{H\min}E_{H})(C_{wf}-C_{L\min}E_{L})[xy(\eta_{t}-1)-\eta_{t}]-C_{L\min}C_{wf}E_{L}T_{L1}x\\ \times y+E_{H}C_{H\min}T_{H1}(C_{wf}-C_{L\min}E_{L})[(\eta_{t}-1)xy-\eta_{t}]\}\{C_{wf}xy(C_{H1}E_{H1}T_{H3}\\ \begin{array}{l} +C_{L\min}E_{L}T_{L1})+\{xy[C_{wf}-C_{H1}E_{H1}+C_{L\min}E_{L}(\eta_{t}-1)]-C_{L\min}E_{L}\eta_{t}\}C_{H\min}\\ \times E_{H}T_{H1}+a_{1}\{C_{L\min}(C_{wf}-E_{H}C_{H\min})E_{L}\{(\eta_{t}-1)xy-\eta_{t}-xy[C_{H\min}C_{wf}E_{H}\\ +C_{H1}E_{H1}(C_{wf}-C_{H\min}E_{H})]\}\}\} \end{array} \end{array}}{C_{wf}^{3}T_{0}xy[a_{1}(C_{wf}-C_{H\min}E_{H})+C_{H\min}E_{H}T_{H1}][(\eta_{t}-1)xy-\eta_{t}]}$$

(33) $$\bar{E}=\frac{\begin{array}{l} \{C_{wf}xy(C_{H1}E_{H1}T_{H3}+C_{L\min}E_{L}T_{L1})+C_{H\min}E_{H}T_{H1}\{xy[C_{wf}-C_{H1}E_{H1}\\ +C_{L\min}E_{L}(\eta_{t}-1)]-C_{L\min}E_{L}\eta_{t}\}+a_{1}\{C_{L\min}E_{L}(C_{wf}-C_{H\min}E_{H})[(\eta_{t}\\ -1)xy-\eta_{t}]-xy[C_{wf}C_{H\min}E_{H}+C_{H1}E_{H1}(C_{wf}-C_{H\min}E_{H})]\}\}/(T_{0}\\ \begin{array}{l} \times xy)-C_{wf}\{C_{L}\ln\{1+\{C_{L\min}E_{L}\{a_{1}C_{wf}\eta_{t}-C_{wf}xy[a_{1}(\eta_{t}-1)+T_{L1}]\\ +C_{H\min}E_{H}(a_{1}-T_{H1})[(\eta_{t}-1)xy-\eta_{t}]\}\}/(C_{L}C_{wf}T_{L1}xy)\}+C_{H}\ln\{[a_{1}\\ \times C_{H\min}E_{H}+(C_{H}-C_{H\min}E_{H})T_{H1}]/(C_{H}T_{H1})\}+C_{H1}\ln\{1+\{E_{H1}[C_{wf}\\ \times (a_{1}-T_{H3})+E_{H}C_{H\min}(T_{H1}-a_{1})]\}/(C_{wf}T_{H3})\}\} \end{array} \end{array}}{C_{wf}^{2}}$$

where

(34) $$a_{1}=\frac{\left(\eta_{c}+x-1)\{C_{Lmin}C_{wf}E_{L}T_{L1}xy-C_{Hmin}E_{H}T_{H1}(C_{wf}-C_{Lmin}E_{L})[(\eta_{t}-1)xy-\eta_{t}]\right\}}{\begin{array}{l} C_{Hmin}C_{Lmin}E_{H}E_{L}(\eta_{c}+x-1)(\eta_{t}xy-xy-\eta_{t})+C_{wf}^{2}[xy-x^{2}y+\eta_{t}(\eta_{c}+x\\ -1)(xy-1)]-C_{wf}(\eta_{c}+x-1)(E_{H}C_{Hmin}+E_{L}C_{Lmin})[(\eta_{t}-1)xy-\eta_{t}] \end{array}}$$

Parameters x and y in Equations (30)–(34) can be obtained by Equations (13) and (19), and then the arithmetic solution of $\bar{W}$, η, $\bar{P}$ and $\bar{E}$ can be gained. When $C_{H}$, $C_{H1}$, $C_{L}$, $E_{H}$, $E_{H1}$, $E_{L}$, $\eta_{c}$ and $\eta_{t}$ are specific values, the cycle could be transformed into different cycle models. Equations (30)–(34) could be simplified into the performance indicators of the various cycle models, which have certain universality.

**Figure 3:**
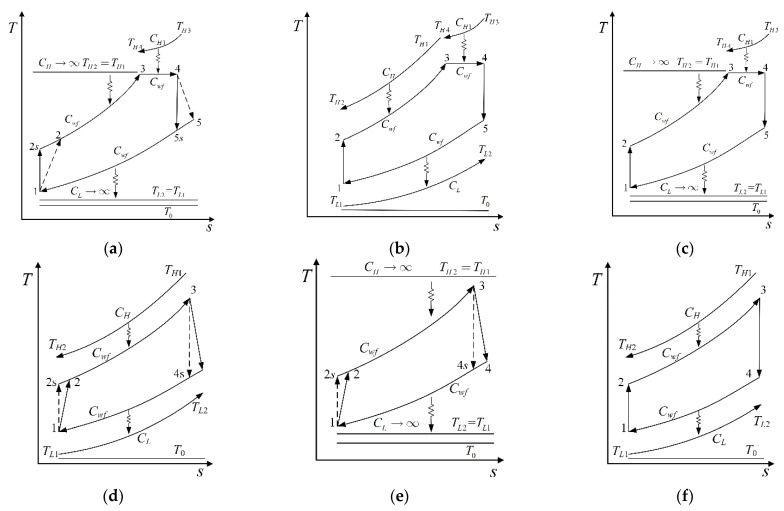

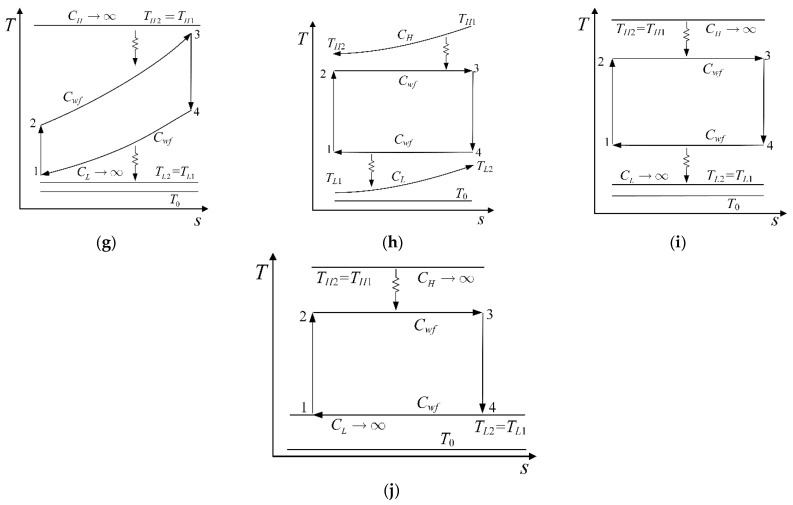
Diagrams of (**a**) irreversible simple BCY with an IHP and coupled to CTHRs; (**b**) endoreversible simple BCY with an IHP and coupled to VTHRs; (**c**) endoreversible simple BCY with an IHP and coupled to CTHRs; (**d**) simple irreversi-ble BCY coupled to VTHRs; (**e**) simple irreversible BCY coupled to CTHRs; (**f**) simple endoreversible BCY coupled to VTHRs; (**g**) simple endoreversible BCY coupled to CTHRs; (**h**) endoreversible Carnot cycle coupled to VTHRs; (**i**) endoreversible Carnot cycle coupled to CTHRs; (**j**) endoreversible Novikov cycle coupled to CTHRs.

1. When $C_{H1}=C_{L}\to \infty$, Equations (30)–(34) can be simplified into the performance indicators of the irreversible simple BCY with an IHP and coupled to constant-temperature heat reservoirs (CTHRs) whose T−s diagram is shown in Figure 3a:
  (35) $$\bar{W}=\frac{\begin{array}{l} C_{wf}xy(C_{H1}E_{H1}T_{H3}+C_{L\min}E_{L}T_{L1})+C_{H\min}E_{H}T_{H1}\{xy[C_{wf}\\ -C_{H1}E_{H1}+C_{L\min}E_{L}(\eta_{t}-1)]-C_{L\min}E_{L}\eta_{t}\}+a_{1}\{C_{L\min}E_{L}\\ \times [(\eta_{t}-1)xy-\eta_{t}](C_{wf}-E_{H}C_{H\min})-xy[C_{wf}C_{H\min}E_{H}\\ +C_{H1}E_{H1}(C_{wf}-C_{H\min}E_{H})]\} \end{array}}{C_{wf}^{2}T_{0}xy}$$
  (36) $$\eta =\frac{\begin{array}{l} C_{wf}E_{H}E_{L}\eta_{t}T_{H1}-\{E_{H}T_{H1}[C_{wf}-C_{H1}E_{H1}+C_{wf}E_{L}(\eta_{t}-1)]+(C_{H1}E_{H1}T_{H3}\\ +C_{wf}E_{L}T_{L1})\}xy+a_{2}\{[C_{wf}E_{H}+C_{H1}E_{H1}(1-E_{H})]xy-C_{wf}E_{L}(1-E_{H})\\ \times [-\eta_{t}+(-1+\eta_{t})xy]\} \end{array}}{xy\{a_{2}[C_{H1}E_{H1}+E_{H}(C_{wf}-C_{H1}E_{H1})]+E_{H}T_{H1}(C_{H1}E_{H1}-C_{wf})-C_{H1}E_{H1}T_{H3}\}}$$
  (37) $$\bar{P}=\frac{\begin{array}{l} C_{wf}\{-E_{L}T_{L1}xy+a_{2}(1-E_{H})(1-E_{L})[(\eta_{t}-1)xy-\eta_{t}]+E_{H}T_{H1}(1-E_{L})[(\eta_{t}\\ -1)xy-\eta_{t}]\}\{xy(C_{H1}E_{H1}T_{H3}+C_{wf}E_{L}T_{L1})+E_{H}T_{H1}\{xy[C_{wf}-C_{H1}E_{H1}+C_{wf}\\ \times E_{L}(\eta_{t}-1)]-C_{wf}E_{L}\eta_{t}\}+a_{2}C_{wf}(1-E_{H})E_{L}\{(\eta_{t}-1)xy-\eta_{t}-C_{wf}xy[C_{wf}E_{H}\\ +C_{H1}E_{H1}(1-E_{H})]\}\} \end{array}}{C_{wf}^{3}T_{0}xy[a_{2}(1-E_{H})+E_{H}T_{H1}][(\eta_{t}-1)xy-\eta_{t}]}$$
  (38) $$\bar{E}=\frac{\begin{array}{l} \left\{xy(C_{H1}E_{H1}T_{H3}+C_{wf}E_{L}T_{L1})+E_{H}T_{H1}\{[C_{wf}-C_{H1}E_{H1}+C_{wf}E_{L}(\eta_{t}-1)]xy-E_{L}\eta_{t}\right\}\\ +a_{2}C_{wf}\{C_{wf}E_{L}(1-E_{H})[(\eta_{t}-1)xy-\eta_{t}]-xy[C_{wf}E_{H}+C_{H1}(1-E_{H})E_{H1}]\}\}/(T_{0}xy)\\ \begin{array}{l} -\{C_{H}\ln[(a_{2}C_{wf}E_{H}+C_{H}T_{H1}-C_{wf}E_{H}T_{H1})/(C_{H}T_{H1})]+C_{H1}\ln\{1+\{E_{H1}[a_{2}+C_{wf}E_{H}\\ \times (T_{H1}-a_{2})/C_{wf}-T_{H3}]\}/T_{H3}\}+C_{L}\ln\{1+\{C_{wf}E_{L}\{a_{2}\eta_{t}-xy[a_{2}(\eta_{t}-1)+T_{L1}]+E_{H}\\ \times (a_{2}-T_{H1})[(\eta_{t}-1)xy-\eta_{t}]\}\}/(C_{L}T_{L1}xy)\}\} \end{array} \end{array}}{C_{wf}}$$
  where
  (39) $$a_{2}=\frac{\left(\eta_{c}+x-1)\{-E_{L}T_{L1}xy-E_{H}T_{H1}(1-E_{L})[(\eta_{t}-1)xy-\eta_{t}]\right\}}{\begin{array}{l} E_{H}E_{L}(\eta_{c}+x-1)[(xy-1)\eta_{t}-xy]+[xy-x^{2}y+\eta_{t}(\eta_{c}+x-1)\\ \times (xy-1)]-[(\eta_{t}-1)xy-\eta_{t}](\eta_{c}+x-1)(E_{H}+E_{L}) \end{array}}$$
2. When $\eta_{c1}=\eta_{t1}=1$, Equations (30)–(34) can be respectively simplified into the performance indicators of the endoreversible simple BCY with an IHP and coupled to VTHRs [99], whose T−s diagram is shown in Figure 3b:
  (40) $$\bar{W}=\frac{\begin{array}{l} C_{wf}x\{C_{L\min}C_{wf}E_{L}T_{L1}(y-1)+C_{H1}E_{H1}[C_{wf}T_{H3}(y-1)+C_{L\min}E_{L}(T_{H3}\\ -T_{L1}xy)]\}+E_{H}C_{H\min}\{C_{L\min}E_{L}[C_{wf}T_{H1}(x-1)+C_{wf}T_{L1}x(1-xy)+C_{H1}\\ \times E_{H1}x(T_{L1}xy-T_{H3})]+xC_{wf}[(y-1)C_{wf}T_{H1}+C_{H1}E_{H1}(T_{H3}-T_{H1}y)]\} \end{array}}{C_{wf}T_{0}x[C_{wf}^{2}y-(C_{wf}-C_{H\min}E_{H})(C_{wf}-C_{L\min}E_{L})]}$$
  (41) $$\eta =\frac{\begin{array}{l} C_{wf}T_{0}x\{C_{L\min}C_{wf}E_{L}T_{L1}(y-1)+C_{H1}E_{H1}[C_{wf}T_{H3}(y-1)+C_{L\min}E_{L}(T_{H3}-T_{L1}xy)]\}\\ \begin{array}{l} +C_{H\min}E_{H}\{C_{L\min}E_{L}[C_{wf}T_{H1}(x-1)+C_{wf}T_{L1}x(1-xy)+C_{H1}E_{H1}x(T_{L1}xy-T_{H3})]\\ +C_{wf}x[C_{wf}T_{H1}(y-1)+C_{H1}E_{H1}(T_{H3}-T_{H1}y)]\} \end{array} \end{array}}{\begin{array}{l} C_{wf}T_{0}x\{C_{H\min}E_{H}[C_{wf}^{2}T_{H1}(y-1)+C_{H1}C_{wf}E_{H1}(T_{H3}-T_{H1}y)+C_{L\min}C_{wf}E_{L}(T_{H1}-T_{L1}xy)\\ +C_{H1}C_{L\min}E_{H1}E_{L}(T_{L1}xy-T_{H3})]+C_{H1}C_{wf}E_{H1}[C_{wf}T_{H3}(y-1)+C_{L\min}E_{L}(T_{H3}-T_{L1}xy)]\} \end{array}}$$
  (42) $$\bar{P}=\frac{\begin{array}{l} {}[C_{H\min}E_{H}T_{H1}(C_{wf}-C_{L\min}E_{L})+C_{L\min}C_{wf}E_{L}T_{L1}xy]\{C_{wf}x\{C_{L\min}C_{wf}E_{L}T_{L1}(y-1)+C_{H1}E_{H1}\\ \begin{array}{l} \times [C_{wf}T_{H3}(y-1)+C_{L\min}E_{L}(T_{H3}-T_{L1}xy)]\}+C_{H\min}E_{H}\{C_{wf}x[C_{wf}T_{H1}(y-1)+(T_{H3}-T_{H1}\\ \times y)C_{H1}E_{H1}]+C_{L\min}E_{L}[C_{wf}T_{H1}(x-1)+C_{wf}T_{L1}x(1-xy)+C_{H1}E_{H1}x(T_{L1}xy-T_{H3})]\}\} \end{array} \end{array}}{\begin{array}{l} C_{wf}T_{0}x[C_{wf}^{2}y-(C_{wf}-C_{H\min}E_{H})(C_{wf}-C_{L\min}E_{L})][C_{L\min}(C_{wf}-C_{H\min}E_{H})E_{L}\\ \times T_{L1}x+C_{H\min}C_{wf}E_{H}T_{H1}] \end{array}}$$
  (43) $$\bar{E}=\frac{\begin{array}{l} C_{wf}x\{C_{L\min}C_{wf}E_{L}T_{L1}(y-1)+C_{H1}E_{H1}[C_{wf}T_{H3}(y-1)+C_{L\min}E_{L}(T_{H3}\\ -T_{L1}xy)]\}+C_{H\min}E_{H}\{C_{L\min}E_{L}[C_{wf}T_{H1}(x-1)+C_{wf}T_{L1}x(1-xy)+C_{H1}\\ \times E_{H1}x(T_{L1}xy-T_{H3})]+C_{wf}x[C_{wf}T_{H1}(y-1)+(T_{H3}-T_{H1}y)C_{H1}E_{H1}]\} \end{array}}{\begin{array}{c} C_{wf}T_{0}x[C_{wf}^{2}y-(C_{wf}-C_{H\min}E_{H})(C_{wf}-C_{L\min}E_{L})]\\ -\frac{C_{H}}{C_{wf}T_{0}}\ln\{1+\frac{\begin{array}{l} C_{H\min}C_{wf}E_{H}(C_{wf}T_{H1}-C_{L\min}E_{L}T_{H1}-C_{wf}T_{H1}y+C_{L\min}\\ \times E_{L}T_{L1}xy) \end{array}}{T_{H1}[C_{H}C_{wf}^{2}y-C_{H}(C_{wf}-C_{H\min}E_{H})(C_{wf}-C_{L\min}E_{L})]}\}\\ -\frac{C_{H1}}{C_{wf}T_{0}}\ln\frac{\begin{array}{l} \{(C_{wf}-C_{H\min}E_{H})(E_{H1}-1)(C_{wf}-C_{L\min}E_{L})T_{H3}+C_{wf}y\\ \times [C_{H\min}E_{H}E_{H1}T_{H1}-C_{wf}(E_{H1}-1)T_{H3}]+C_{L\min}E_{H1}E_{L}T_{L1}xy\\ \times (C_{wf}-C_{H\min}E_{H})\} \end{array}}{C_{wf}^{2}T_{H3}y-T_{H3}(C_{wf}-C_{H\min}E_{H})(C_{wf}-C_{L\min}E_{L})}\\ -\frac{C_{L}}{C_{wf}T_{0}}\ln\{1+\frac{C_{L\min}C_{wf}E_{L}[C_{H\min}E_{H}(T_{H1}-T_{L1}x)-C_{wf}T_{L1}x(y-1)]}{C_{L}T_{L1}[(C_{H\min}E_{H}-C_{wf})(C_{wf}C_{L\min}E_{L})x+C_{wf}^{2}xy]}\} \end{array}}$$
3. When $\eta_{c1}=\eta_{t1}=1$ and $C_{H1}=C_{H2}=C_{L}\to \infty$, Equations (30)–(34) can be simplified into the performance indicators of the endoreversible simple BCY with an IHP and coupled to CTHRs, whose T−s diagram is shown in Figure 3c:
  (44) $$\bar{W}=\frac{\begin{array}{l} C_{wf}x\{C_{wf}E_{L}T_{L1}(y-1)+C_{H1}E_{H1}[E_{L}T_{H3}-E_{L}T_{L1}xy+T_{H3}(y-1)]\}\\ +C_{wf}E_{H}\{E_{L}[T_{H1}C_{wf}(x-1)+C_{wf}T_{L1}x(1-xy)+C_{H1}E_{H1}x(T_{L1}xy\\ -T_{H3})]+x[C_{wf}T_{H1}(y-1)+C_{H1}E_{H1}(T_{H3}-T_{H1}y)]\} \end{array}}{C_{wf}^{2}T_{0}x(E_{H}+E_{L}+y-E_{H}E_{L}-1)}$$
  (45) $$\eta =\frac{\begin{array}{l} T_{0}x\{C_{wf}E_{L}T_{L1}y-C_{wf}E_{L}T_{L1}+C_{H1}E_{H1}[T_{H3}y-T_{H3}+T_{H3}E_{L}-E_{L}T_{L1}xy]\}\\ \begin{array}{l} +\{E_{H}\{x[C_{wf}T_{H1}y-C_{wf}T_{H1}+C_{H1}E_{H1}(T_{H3}-T_{H1}y)]+E_{L}[C_{wf}T_{H1}x-C_{wf}\\ \times T_{H1}+C_{wf}T_{L1}x(1-xy)+C_{H1}E_{H1}x(-T_{H3}+T_{L1}xy)]\} \end{array} \end{array}}{\begin{array}{l} C_{wf}T_{0}x\{[C_{wf}T_{H1}y-C_{wf}T_{H1}+C_{H1}E_{H1}(T_{H3}-T_{H1}y)+C_{wf}E_{L}(T_{H1}-T_{L1}xy)\\ +C_{H1}E_{H1}E_{L}(T_{L1}xy-T_{H3})]E_{H}+C_{H1}E_{H1}[T_{H3}(y-1)+E_{L}(T_{H3}-T_{L1}xy)]\} \end{array}}$$
  (46) $$\bar{P}=\frac{\begin{array}{l} \left[E_{H}T_{H1}(1-E_{L})+E_{L}T_{L1}xy]\{C_{wf}x\{C_{wf}E_{L}T_{L1}(y-1)+C_{H1}E_{H1}[T_{H3}(y-1\right)\\ +E_{L}(T_{H3}-T_{L1}xy)]\}+E_{H}C_{wf}\{x[C_{wf}T_{H1}(y-1)+C_{H1}E_{H1}(T_{H3}-T_{H1}y)]\\ +E_{L}[C_{wf}T_{H1}(x-1)+C_{wf}T_{L1}x(1-xy)+C_{H1}xE_{H1}(T_{L1}xy-T_{H3})]\}\} \end{array}}{C_{wf}^{2}T_{0}x(E_{H}+E_{L}+y-E_{H}E_{L}-1)(E_{H}T_{H1}+E_{L}T_{L1}x-E_{L}T_{L1}xE_{H})}$$
  (47) $$\bar{E}=\frac{\begin{array}{l} \{C_{wf}E_{L}T_{L1}(y-1)+C_{H1}E_{H1}[T_{H3}(y-1)+E_{L}T_{H3}T_{L1}xy]\}x\\ +E_{H}\{E_{L}[C_{wf}T_{H1}(x-1)+(1-xy)C_{wf}T_{L1}x+C_{H1}E_{H1}x(T_{L1}\\ \times xy-T_{H3})]+x[C_{wf}T_{H1}(y-1)+C_{H1}E_{H1}(T_{H3}-T_{H1}y)]\} \end{array}}{\begin{array}{c} C_{wf}T_{0}xy-C_{wf}T_{0}x(1-E_{H}-E_{L}+E_{H}E_{L})\\ -\frac{C_{H1}}{C_{wf}T_{0}}\ln\frac{\begin{array}{l} C_{wf}T_{H3}(1-E_{H}-E_{L}+E_{H}E_{L})(E_{H1}-1)+C_{wf}\\ \times y[E_{H}E_{H1}T_{H1}-T_{H3}(E_{H1}-1)]+E_{H1}E_{L}T_{L1}xy\\ \times (1-E_{H}) \end{array}}{C_{wf}[T_{H3}y-T_{H3}(1-E_{H})(1-E_{L})]}\\ -\frac{C_{H}}{C_{wf}T_{0}}\ln\{1+\frac{E_{H}C_{wf}(T_{H1}-E_{L}\times T_{H1}-T_{H1}y+E_{L}T_{L1}xy)}{T_{H1}C_{H}[y-(1-E_{H})(1-E_{L})]}\}\\ -\frac{C_{L}}{C_{wf}T_{0}}\ln\{1+\frac{E_{L}C_{wf}[E_{H}(T_{H1}-T_{L1}x)-T_{L1}x(y-1)]}{C_{L}T_{L1}[(E_{H}-1)(1-E_{L})x+xy]}\} \end{array}}$$
4. When $E_{H1}=0$, Equations (30)–(34) can be simplified into the performance indicators of the simple irreversible BCY coupled to VTHRs [79], whose T−s diagram is shown in Figure 3d:
  (48) $$\bar{W}=\frac{\begin{array}{l} C_{L\min}C_{wf}E_{L}T_{L1}x+C_{H\min}E_{H}T_{H1}\{C_{L\min}E_{L}[\eta_{t}(x-1)-x]+C_{wf}x\}\\ +a_{3}\{C_{L\min}(C_{wf}-C_{H\min}E_{H})E_{L}[\eta_{t}(x-1)-x]-C_{H\min}C_{wf}E_{H}x\} \end{array}}{C_{wf}^{2}T_{0}x}$$
  (49) $$\eta =\frac{\begin{array}{l} a_{3}\{C_{H\min}C_{wf}E_{H}x-C_{L\min}E_{L}(C_{wf}-C_{H\min}E_{H})[\eta_{t}(x-1)-x]\}-C_{L\min}\\ \times C_{wf}E_{L}T_{L1}x+C_{H\min}E_{H}T_{H1}[C_{L\min}E_{L}(\eta_{t}+x-\eta_{t}x)-C_{wf}x] \end{array}}{xC_{H\min}E_{H}C_{wf}(a_{3}-T_{H1})}$$
  (50) $$\bar{P}=\frac{\begin{array}{l} \left\{-a_{3}[\eta_{t}(x-1)-x](C_{wf}-C_{H\min}E_{H})(C_{wf}-C_{L\min}E_{L})-C_{H\min}E_{H}T_{H1}[\eta_{t}(x-1)-x\right]\\ \times (C_{wf}-C_{L\min}E_{L})+C_{L\min}C_{wf}E_{L}T_{L1}x\}\{a_{3}\{C_{L\min}E_{L}[\eta_{t}(x-1)-x](C_{wf}-C_{H\min}E_{H})\\ -C_{H\min}C_{wf}E_{H}x\}+C_{H\min}E_{H}T_{H1}\{C_{L\min}E_{L}[\eta_{t}(x-1)-x]+C_{wf}x\}+C_{L\min}C_{wf}E_{L}T_{L1}x\} \end{array}}{-C_{wf}^{3}T_{0}x[\eta_{t}(x-1)-x][a_{3}(C_{wf}-C_{H\min}E_{H})+C_{H\min}E_{H}T_{H1}]}$$
  (51) $$\bar{E}=\frac{\begin{array}{l} \{C_{L\min}C_{wf}E_{L}T_{L1}x+C_{H\min}E_{H}T_{H1}\{C_{L\min}E_{L}[\eta_{t}(x-1)-x]+C_{wf}x\}+a_{3}\{C_{L\min}(C_{wf}\\ -C_{H\min}E_{H})E_{L}[\eta_{t}(x-1)-x]-C_{H\min}C_{wf}E_{H}x\}\}/(T_{0}x)-C_{wf}\{C_{H}\ln[1+C_{H\min}E_{H}\\ \times (a_{3}-T_{H1})/(C_{H}T_{H1})]+C_{L}\ln\{1+C_{L\min}E_{L}\{a_{3}C_{wf}\eta_{t}+C_{H\min}E_{H}(a_{3}-T_{H1})[\eta_{t}(x-1)\\ -x]-C_{wf}[a_{3}(\eta_{t}-1)+T_{L1}]x\}/(C_{L}C_{wf}T_{L1}x)\}\} \end{array}}{C_{wf}^{2}}$$
  where
  (52) $$a_{3}=\frac{\left(\eta_{c}+x-1)\{C_{Lmin}C_{wf}E_{L}T_{L1}x-C_{Hmin}E_{H}T_{H1}(C_{wf}-C_{Lmin}E_{L})[(\eta_{t}-1)x-\eta_{t}]\right\}}{\begin{array}{l} C_{Hmin}C_{Lmin}E_{H}E_{L}(\eta_{c}+x-1)(\eta_{t}x-x-\eta_{t})+C_{wf}^{2}[x-x^{2}+\eta_{t}(\eta_{c}+x-1)(x\\ -1)]-C_{wf}(\eta_{c}+x-1)(E_{H}C_{Hmin}+E_{L}C_{Lmin})\times [(\eta_{t}-1)x-\eta_{t}] \end{array}}$$
5. When $E_{H1}=0$ and $C_{H}=C_{L}\to \infty$, Equations (30)–(34) can be simplified into the performance indicators of the simple irreversible BCY coupled to CTHRs [76], whose T−s diagram is shown in Figure 3e:
  (53) $$\bar{W}=\frac{E_{L}T_{L1}x-a_{4}\{(E_{H}-1)E_{L}[\eta_{t}(x-1)-x]+E_{H}x\}+E_{H}T_{H1}[E_{L}\eta_{t}(x-1)+x-E_{L}x]}{T_{0}x}$$
  (54) $$\eta =\frac{\begin{array}{l} a_{4}(E_{H}-1)E_{L}[\eta_{t}(x-1)-x]+a_{4}E_{H}x-E_{H}T_{H1}x-E_{L}T_{L1}x\\ +E_{H}E_{L}T_{H1}(\eta_{t}+x-\eta_{t}x) \end{array}}{xE_{H}(a_{4}-T_{H1})}$$
  (55) $$\bar{P}=\frac{\begin{array}{l} \{a_{4}(E_{H}-1)(E_{L}-1)[\eta_{t}(x-1)-x]-E_{H}T_{H1}(E_{L}-1)[\eta_{t}(x\\ -1)-x]-E_{L}T_{L1}x\}\{a_{4}(E_{H}-1)E_{L}[\eta_{t}(x-1)-x]+a_{4}E_{H}x\\ -E_{H}T_{H1}x-E_{L}T_{L1}x+E_{H}E_{L}T_{H1}(\eta_{t}+x-\eta_{t}x)\} \end{array}}{T_{0}[a_{4}(E_{H}-1)-E_{H}T_{H1}][\eta_{t}(x-1)-x]x}$$
  (56) $$\begin{array}{l} \bar{E}=\{E_{L}T_{L1}x-a_{4}\{E_{L}(E_{H}-1)[\eta_{t}(x-1)-x]+E_{H}x\}+E_{H}T_{H1}[E_{L}\eta_{t}(x\\ -1)+x-E_{L}x]\}/(T_{0}x)-C_{H}\ln[1+C_{wf}E_{H}(a_{4}-T_{H1})/(C_{H}T_{H1})]/C_{wf}\\ -C_{L}\ln\{1+C_{wf}E_{L}\{a_{4}(E_{H}-1)[\eta_{t}(x-1)-x]-T_{L1}x+E_{H}T_{H1}(\eta_{t}+x\\ -\eta_{t}x)\}/(C_{L}T_{L1}x)\}/C_{wf} \end{array}$$
  where
  (57) $$a_{4}=\frac{\left(\eta_{c}+x-1)E_{H}T_{H1}(E_{L}-1)[\eta_{t}(x-1)-x]+E_{L}T_{L1}x\right\}}{\left(E_{H}-1)(E_{L}-1)(x-1)(\eta_{c}+x-1)\eta_{t}-x[x-1+E_{H}(E_{L}-1)(\eta_{c}+x-1)-E_{L}(\eta_{c}+x-1)\right]}$$
6. When $E_{H1}=0$ and $\eta_{c}=\eta_{t}=1$, Equations (30)–(34) can be simplified into the performance indicators of the simple endoreversible BCY coupled to VTHRs [78], whose T−s diagram is shown in Figure 3f:
  (58) $$\bar{W}=\frac{C_{H\min}C_{L\min}E_{H}E_{L}(-1+x)(T_{H1}-T_{L1}x)}{T_{0}x[C_{L\min}C_{wf}E_{L}+C_{H\min}E_{H}(C_{wf}-C_{L\min}E_{L})]}$$
  (59) η=(x−1)/x
  (60) $$\bar{P}=\frac{\begin{array}{l} C_{H\min}C_{L\min}E_{H}E_{L}(-1+x)(T_{H1}-T_{L1}x)[C_{H\min}E_{H}(C_{wf}\\ -C_{L\min}E_{L})T_{H1}+C_{L\min}C_{wf}E_{L}T_{L1}x] \end{array}}{\begin{array}{l} T_{0}x[C_{L\min}C_{wf}E_{L}+C_{H\min}E_{H}(C_{wf}-C_{L\min}E_{L})][C_{L\min}C_{wf}\\ \times E_{L}T_{L1}x+C_{H\min}E_{H}(C_{wf}T_{H1}-C_{L\min}E_{L}T_{L1}x)] \end{array}}$$
  (61) $$\bar{E}=\frac{\begin{array}{l} \frac{C_{Hmin}C_{Lmin}C_{wf}E_{H}E_{L}(x-1)(T_{H1}-T_{L1}x)}{\begin{array}{l} {}[C_{Lmin}C_{wf}E_{L}+C_{Hmin}E_{H}(C_{wf}\\ -C_{Lmin}EL)]T_{0}x \end{array}}-C_{H}\ln[1+\frac{C_{Hmin}C_{Lmin}C_{wf}E_{H}E_{L}(T_{L1}x-T_{H1})}{\begin{array}{l} C_{H}[C_{Lmin}C_{wf}E_{L}+C_{Hmin}E_{H}\\ (C_{wf}-C_{Lmin}E_{L})]T_{H1} \end{array}}]\\ -C_{L}\ln\{\frac{C_{L}C_{Lmin}C_{wf}E_{L}T_{L1}x+C_{Hmin}E_{H}[C_{L}C_{wf}T_{L1}x+C_{Lmin}E_{L}(C_{wf}T_{H1}-C_{L}T_{L1}x-C_{wf}T_{L1}x)]}{C_{L}[C_{Lmin}C_{wf}E_{L}+C_{Hmin}E_{H}(C_{wf}-C_{Lmin}E_{L})]T_{L1}x}\} \end{array}}{C_{wf}}$$
7. When $E_{H1}=0$, $\eta_{c}=\eta_{t}=1$ and $C_{H}=C_{L}\to \infty$, Equations (30)–(34) can be simplified into the performance indicators of the simple endoreversible BCY coupled to CTHRs [77], whose T−s diagram is shown in Figure 3g:
  (62) $$\bar{W}=\frac{E_{H}E_{L}(-1+x)(T_{L1}x-T_{H1})}{[E_{H}(E_{L}-1)-E_{L}]T_{0}x}$$
  (63) η=(x−1)/x
  (64) $$\bar{P}=\frac{E_{H}E_{L}(x-1)(T_{L1}x-T_{H1})[E_{H}(E_{L}-1)T_{H1}-E_{L}T_{L1}x]}{T_{0}x(E_{H}E_{L}T_{L1}x-E_{H}T_{H1}-E_{L}T_{L1}x)[E_{H}(E_{L}-1)-E_{L}]}$$
  (65) $$\bar{E}=\frac{\begin{array}{l} C_{wf}E_{H}E_{L}(x-1)(T_{L1}x-T_{H1})+C_{H}T_{0}x(E_{H}+E_{L}-E_{H}E_{L})\ln\{1-C_{wf}E_{H}\\ \times E_{L}(T_{H1}-T_{L1}x)/[C_{H}T_{H1}(E_{H}+E_{L}-E_{H}E_{L})]\}+C_{L}T_{0}x(E_{H}+E_{L}-E_{H}\\ \times E_{L})\ln[1+C_{wf}E_{H}E_{L}(T_{H1}-T_{L1}x)/(C_{L}(E_{H}+E_{L}-E_{H}E_{L})T_{L1}x)] \end{array}}{C_{wf}[E_{H}(E_{L}-1)-E_{L}]T_{0}x}$$
8. When $E_{H}=E_{L}=0$, $\eta_{c}=\eta_{t}=1$ and $C_{wf}\to \infty$, the cycle in this paper can become the endoreversible Carnot cycle coupled to VTHRs [14], whose T−s diagram is shown in Figure 3h. However, Equations (30), (33), and (34) need to be de-dimensionalized to simplify to W, P and E of the endoreversible Carnot cycle coupled to VTHRs. The performance indicators of the cycle are:
  (66) $$W=\frac{C_{H}C_{L}E_{H}E_{L}(x-1)(T_{H1}-T_{L1}x)}{x(C_{H}E_{H}+C_{L}E_{L})}$$
  (67) η=(x−1)/x
  (68) $$P=\frac{C_{H}C_{L}E_{H}E_{L}(x-1)(T_{H1}-T_{L1}x)}{x(C_{H}E_{H}+C_{L}E_{L})}$$
  (69) $$\begin{array}{l} E=\frac{C_{H}C_{L}E_{H}E_{L}(x-1)(T_{H1}-T_{L1}x)}{(C_{H}E_{H}+C_{L}E_{L})x}-C_{H}T_{0}\ln[1+\frac{C_{L}E_{H}E_{L}(T_{L1}x-T_{H1})}{(C_{H}E_{H}+C_{L}E_{L})T_{H1}}]\\ -C_{L}T_{0}\ln[\frac{C_{H}E_{H}E_{L}T_{H1}+C_{H}E_{H}T_{L1}x+C_{L}E_{L}T_{L1}x-C_{H}E_{H}E_{L}T_{L1}x}{C_{H}E_{H}T_{L1}x+C_{L}E_{L}T_{L1}x}] \end{array}$$
9. When $E_{H}=E_{L}=0$, $\eta_{c}=\eta_{t}=1$ and $C_{H1}=C_{L}=C_{wf}\to \infty$, the cycle in this paper can become the endoreversible Carnot cycle coupled to CTHRs [12], whose T−s diagram is shown in Figure 3i. However, Equations (30), (33), and (34) also need to be de-dimensionalized to simplify to W, P and E of the cycle [12,74,82]. The performance indicators of the cycle are:
  (70) $$W=\frac{U_{H}U_{L}(-1+x)(T_{H1}-T_{L1}x)}{(U_{H}+U_{L})x}$$
  (71) η=(x−1)/x
  (72) $$P=\frac{U_{H}U_{L}(-1+x)(T_{H1}-T_{L1}x)}{(U_{H}+U_{L})x}$$
  (73) $$E=\frac{U_{H}U_{L}(T_{H1}-T_{L1}x)[(T_{0}+T_{H1})T_{L1}x-T_{H1}(T_{0}+T_{L1})]}{T_{H1}T_{L1}(U_{H}+U_{L})x}$$
10. When $E_{H}=E_{L}=0$, $\eta_{c}=\eta_{t}=1$, $C_{H1}=C_{L}=C_{wf}\to \infty$, and $U_{L}\to \infty$, the cycle in this paper can become the endoreversible Novikov cycle coupled to CTHRs [11], whose T−s diagram is shown in Figure 3j. However, Equations (30), (33), and (34) also need to be de-dimensionalized to simplify to W, P and E of the cycle [11]. The performance indicators of the cycle are:
  (74) $$W=\frac{U_{H}(x-1)(T_{H1}-T_{L1}x)}{x}$$
  (75) η=(x−1)/x
  (76) $$P=\frac{U_{H}(x-1)(T_{H1}-T_{L1}x)}{x}$$
  (77) $$E=\frac{U_{H}(T_{H1}-T_{L1}x)[T_{H1}T_{L1}(x-1)+T_{0}(T_{L1}x-T_{H1})]}{T_{H1}T_{L1}x}$$
11. Through comparison with the results in Refs [11,12,13,14,59,76,77,78,79,99], it is found that the results of this paper are consistent with those in Refs [11,12,13,14,59,76,77,78,79,99], which further illustrates the accuracy of the model established in this paper. In particular, when the powers in Equations (58), (62), (66), (70), and (74) take the maximum values, namely $x=\sqrt{T_{H1}/T_{L1}}$, the efficiencies at the maximum power point, Equations (59), (63), (67), (71), and (75) are $\eta =1-\sqrt{T_{L1}/T_{H1}}$, which was derived in Refs. [10,11,12] by Moutier [10], Novikov [11], and Curzon and Ahlborn [12]. One can see that the results of this paper include the Novikov–Curzon–Ahlborn efficiency.
12. FTT is the further extension of conventional irreversible thermodynamics. The cycle model established by Curzon and Ahlborn [12] was a reciprocating Carnot cycle, and the finite time was its major feature. The methods used for solving the FTT problem are usually variational principle and optimal control theory. Therefore, such problems of extremal of thermodynamic processes were first named as FTT by Andresen et al. [132] and as Optimization Thermodynamics or Optimal Control in Problems of Extremals of Irreversible Thermodynamic Processes by Orlov and Rudenko [133]. When the research object was extended from reciprocating devices characterized by finite-time to the steady state flow devices characterized by finite-size, one realizes that the physical property of the problems is the heat transfer owing to temperature deference. Therefore, Grazzini [14] termed it Finite Temperature Difference Thermodynamics, and Lu [134] termed it Finite Surface Thermodynamics. In fact, the works performed by Moutier [10] and Novikov [11] were also steady state flow device models. Bejan introduced the effect of temperature difference heat transfer on the total entropy generation of the systems, taking the entropy generation minimization as the optimization objective for designing thermodynamic processes and devices, termed “Entropy Generation Minimization” or “Thermodynamic Optimization” [15,135]. For the steady state flow device models, Feidt [136,137,138,139,140,141,142,143,144,145,146] termed it Finite Physical Dimensions Thermodynamics (FPDT). The model established herein is closer to FPDT. For both reciprocating model and steady state flow model, the suitable name may be thermodynamics of finite size devices and finite time processes, as Bejan termed it [15,135]. According to the idiomatic usage, the theory is termed FTT in this paper.

## 3. Analyses and Optimizations with Each Single Objective

### 3.1. Analyses of Each Single Objective

The impacts of the irreversibility on cycle performance indicators ($\bar{W}$, η, $\bar{P}$ and $\bar{E}$) are analyzed below. In numerical calculations, it is set that $C_{L}=C_{H}=1.2\;\mathrm{kW}/K$, $C_{wf}=1\;\mathrm{kW}/K$, $T_{0}=300\;K$, $C_{H1}=0.6\;\mathrm{kW}/K$, k=1.4, $R_{g}=0.287\;\mathrm{kJ}/(\mathrm{kg}\cdot K)$, $E_{H}=E_{H1}=E_{L}=0.9$, $C_{p}=1.005\;\mathrm{kW}/K$, $\tau_{H1}=4.33$, $\tau_{H3}=5$ and $\tau_{L}=1$. 

**Figure 4:**
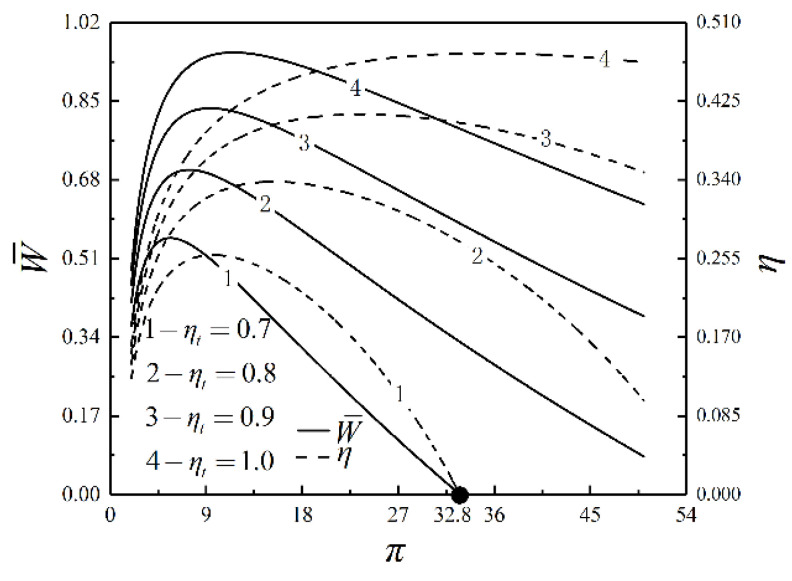
Relationships of $\bar{W}$ and η versus π with different $\eta_{t}$.

**Figure 5:**
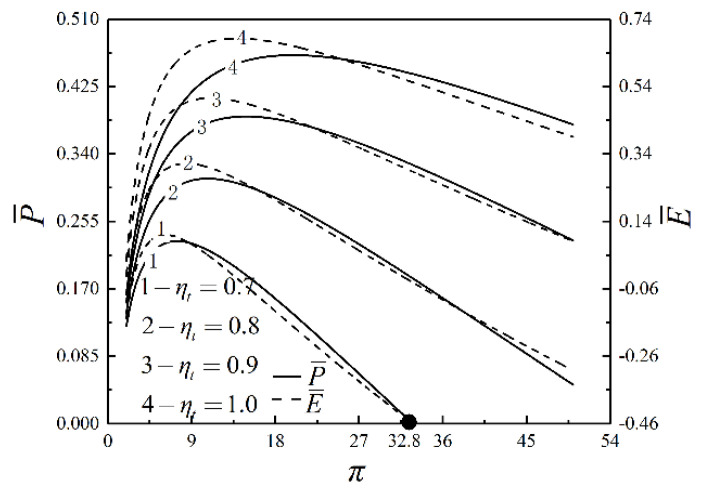
Relationships of $\bar{P}$ and $\bar{E}$ versus π with different $\eta_{t}$.

**Figure 6:**
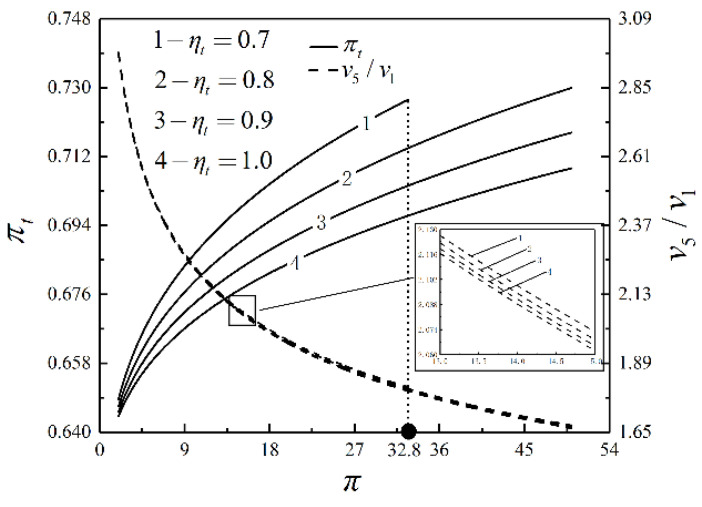
Relationships of $\pi_{t}$ and $v_{5}/v_{1}$ versus π with different $\eta_{t}$.

Figure 4, Figure 5 and Figure 6 present the relationships of $\bar{W}$, η, $\bar{P}$, $\bar{E}$, $\pi_{t}$ and $v_{5}/v_{1}$ versus π with different $\eta_{t}$. As shown in Figure 4 and Figure 5, $\bar{W}$, η, $\bar{P}$ and $\bar{E}$ increase and then decrease as π increases. In the same situation, $\bar{W}$, $\bar{E}$, $\bar{P}$ and η reach the maximum value successively. When $\eta_{t}=0.7$ and π=32.3, $\bar{W}=\bar{P}=0$. If π keeps going up, $\bar{W}$ and $\bar{P}$ are going to go negative. $\bar{W}$, η, $\bar{P}$ and $\bar{E}$ increase as $\eta_{t}$ increases. As π increases, $\bar{W}$, η, $\bar{P}$ and $\bar{E}$ are affected more significantly by $\eta_{t}$. As shown in Figure 6, $\pi_{t}$ goes up but $v_{5}/v_{1}$ goes down as π goes up. $\pi_{t}$ and $v_{5}/v_{1}$ decrease as $\eta_{t}$ rises. It illustrates that the degree of the IHP is improved and the device’s volume is reduced as $\eta_{t}$ increases. 

By numerical calculations, the influences of $\eta_{c}$ on $\bar{W}$, η, $\bar{P}$, $\bar{E}$ and $\pi_{t}$ are the same as those of $\eta_{t}$ on $\bar{W}$, η, $\bar{P}$, $\bar{E}$ and $\pi_{t}$. When $\eta_{t}=0.7$ and π=32.8, $\bar{W}=\bar{P}=0$. However, the impacts of $\eta_{c}$ on $\bar{W}$, η, $\bar{P}$ and $\bar{E}$ are less than those of $\eta_{t}$ on $\bar{W}$, 

η, $\bar{P}$, $\bar{E}$. The effect of $\eta_{c}$ on $\pi_{t}$ is more significant than that of $\eta_{t}$ on $\pi_{t}$. $\eta_{c}$ has little effect on $v_{5}/v_{1}$. In the actual design process, it is suggested that $\eta_{t}$ should be given priority.

**Figure 7:**
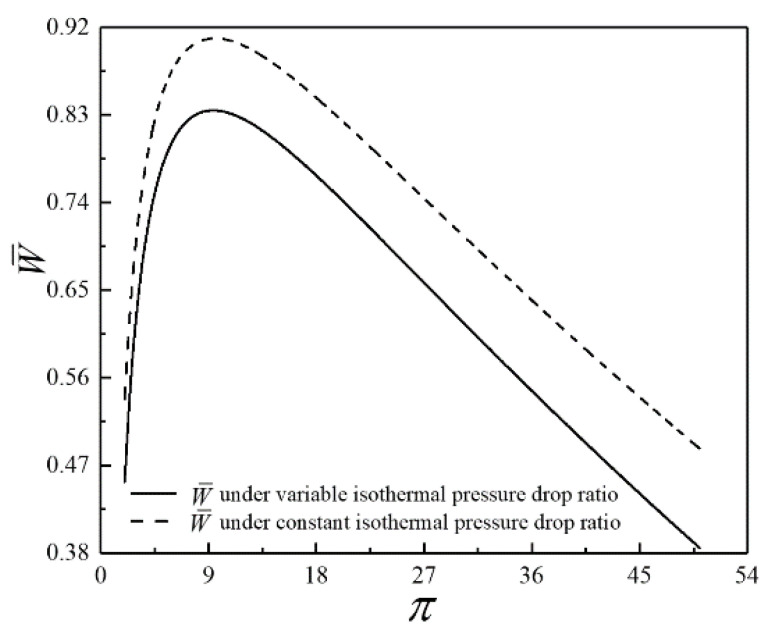
Comparison of $\bar{W}$ under the variable and constant π.

To further explain the difference between the models in this paper and Ref. [101], the comparison of $\bar{W}$ under the variable and constant π is shown in Figure 7. As shown in Figure 7, $\bar{W}$ increases and then decreases as π increases in both cases; that is, the qualitative law is the same. However, there is an apparent quantitative difference between the two points. Under the constant π, $\bar{W}$ corresponding to the constant π is always greater than $\bar{W}$ conforming to the variable π. Similarly, there are quantitative differences in η, $\bar{P}$ and $\bar{E}$ under the variable and constant π. The model whose π is variable is more realistic.

### 3.2. Performance Optimizations for Each Single Objective

With four performance indicators as the OPOs, respectively, the HCDs are optimized under the condition of given total heat conductance ($U_{T}$). The optimal results under different OPOs are compared. The HCDs among the RCC, CCC, and precooler are:

(78) $$u_{H}=U_{H}/U_{T},\;u_{H1}=U_{H1}/U_{T},\;u_{L}=U_{L}/U_{T}$$

The HCDs are must larger than 0, the sum of them is 1, and 2≤π≤50.

**Figure 8:**
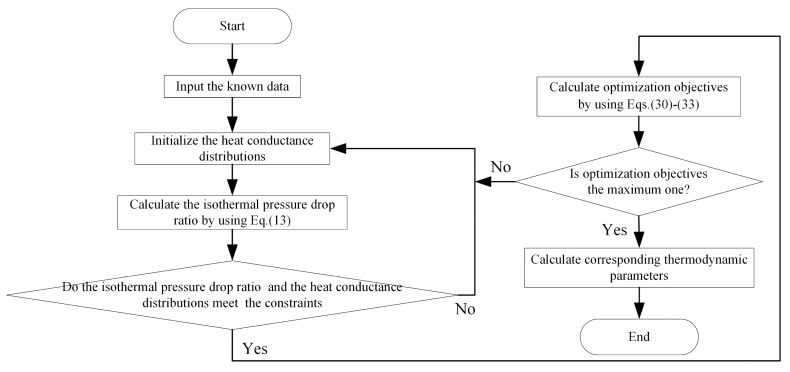
Flowchart of HCD optimization.

Figure 8 shows the flowchart of HCD optimization. The steps are as follows:

1. Enter the known data and the initial values of the HCDs.
2. The $\pi_{t}$ is calculated according to Equation (13).
3. Judge whether the $\pi_{t}$π and HCDs meet the constraints. If they are satisfied, perform step 4; if they are not satisfied, go back to step 1.
4. The performance indicator is solved.
5. Determine whether the inverse objective function is minimized by using the “fmincon” in MATLAB. If it is the smallest, perform step 6; if it is not the slightest, go back to step 1.
6. Calculate the other thermodynamic parameters, and the maximum of the performance indicator is obtained.

#### 3.2.1. Optimizations of Each Single Objective

**Figure 9:**
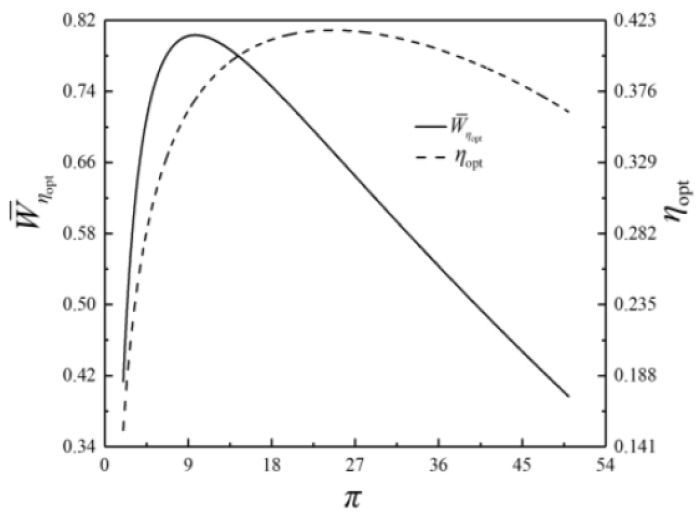
Relationships of ${\bar{W}}_{\eta_{\mathrm{opt}}}$ and $\eta_{\mathrm{opt}}$ versus π.

**Figure 10:**
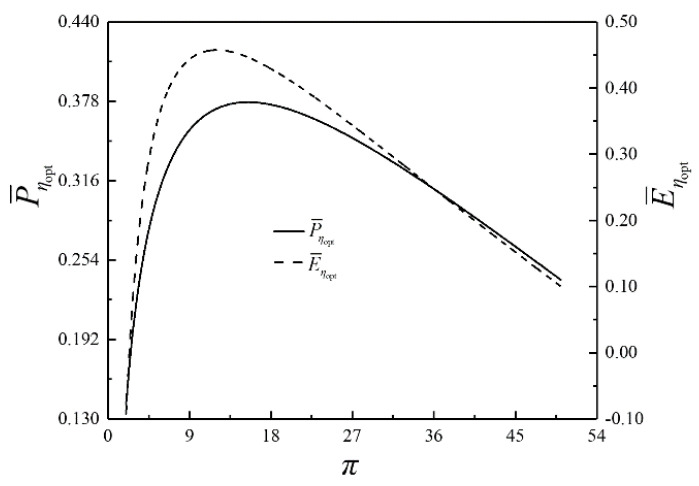
Relationships of ${\bar{P}}_{\eta_{\mathrm{opt}}}$ and ${\bar{E}}_{\eta_{\mathrm{opt}}}$ versus π.

**Figure 11:**
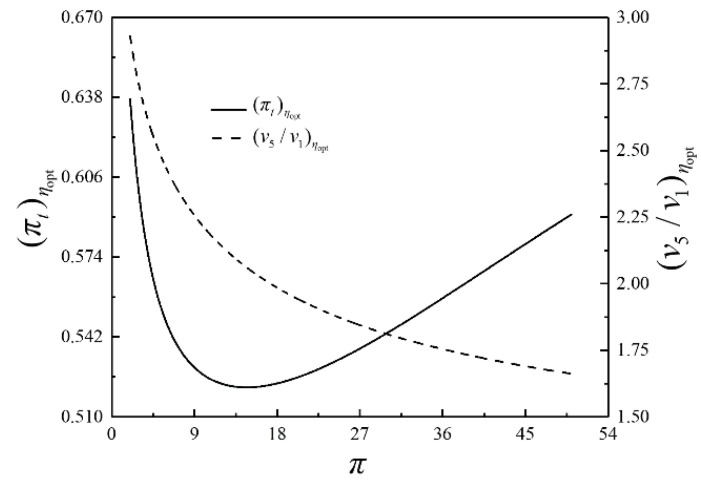
Relationships of ${\left(\pi_{t}\right)}_{\eta_{\mathrm{opt}}}$ and ${\left(v_{5}/v_{1}\right)}_{\eta_{\mathrm{opt}}}$ versus π.

**Figure 12:**
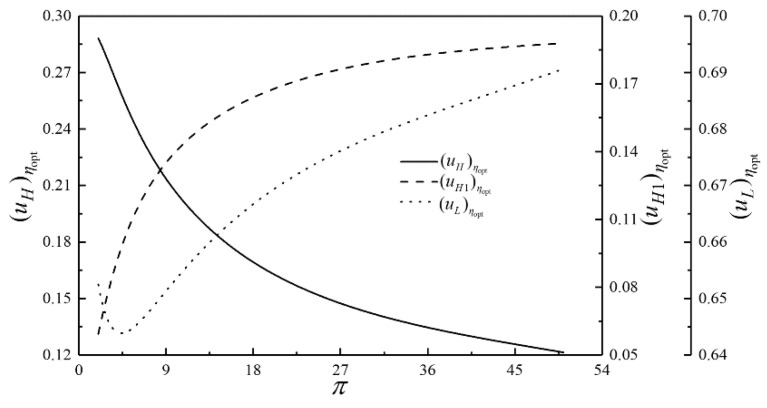
Relationships of ${\left(u_{H}\right)}_{\eta_{\mathrm{opt}}}$, ${\left(u_{H1}\right)}_{\eta_{\mathrm{opt}}}$ and ${\left(u_{L}\right)}_{\eta_{\mathrm{opt}}}$ versus π.

The optimization results of four performance indicators are similar. The optimization results with η as the performance indicator will be mainly discussed herein, while the results with $\bar{W}$, $\bar{P}$ and $\bar{E}$ as the performance indicators are briefly discussed. The relationships of the optimal thermal efficiency ($\eta_{\mathrm{opt}}$) and the corresponding dimensionless power output (${\bar{W}}_{\eta_{\mathrm{opt}}}$) versus π are shown in Figure 9. The relationships of the corresponding dimensionless power density (${\bar{P}}_{\eta_{\mathrm{opt}}}$) and the corresponding dimensionless ecological function (${\bar{E}}_{\eta_{\mathrm{opt}}}$) versus π are demonstrated in Figure 10. As shown in Figure 9 and Figure 10, ${\bar{W}}_{\eta_{\mathrm{opt}}}$, $\eta_{\mathrm{opt}}$, ${\bar{P}}_{\eta_{\mathrm{opt}}}$ and ${\bar{E}}_{\eta_{\mathrm{opt}}}$ first rise and then drops as π rises, which indicates a parabolic relationship with the downward opening. The corresponding isothermal pressure drop ratio (${\left(\pi_{t}\right)}_{\eta_{\mathrm{opt}}}$) and dimensionless maximum specific volume (${\left(v_{5}/v_{1}\right)}_{\eta_{\mathrm{opt}}}$) versus π are shown in Figure 11. ${\left(\pi_{t}\right)}_{\eta_{\mathrm{opt}}}$ decreases and then increases as π increases. It indicates that there is a $\pi_{t}$ that maximizes the degree of isothermal heating in the cycle. ${\left(v_{5}/v_{1}\right)}_{\eta_{\mathrm{opt}}}$ decreases as π increases. The relationships of the HCDs (${\left(u_{H}\right)}_{\eta_{\mathrm{opt}}}$, ${\left(u_{H1}\right)}_{\eta_{\mathrm{opt}}}$ and ${\left(u_{L}\right)}_{\eta_{\mathrm{opt}}}$) versus π are shown in Figure 12. As π increases, ${\left(u_{H}\right)}_{\eta_{\mathrm{opt}}}$ decreases, ${\left(u_{H1}\right)}_{\eta_{\mathrm{opt}}}$ increases rapidly and then slowly, and ${\left(u_{L}\right)}_{\eta_{\mathrm{opt}}}$ decreases first and then increases gradually.

By numerical calculations, ${\bar{W}}_{\mathrm{opt}}$, $\eta_{{\bar{W}}_{\mathrm{opt}}}$, ${\bar{P}}_{{\bar{W}}_{\mathrm{opt}}}$, ${\bar{E}}_{{\bar{W}}_{\mathrm{opt}}}$,${\bar{W}}_{{\bar{P}}_{\mathrm{opt}}}$,.$\eta_{{\bar{P}}_{\mathrm{opt}}}$., ${\bar{P}}_{\mathrm{opt}}$, ${\bar{E}}_{{\bar{P}}_{\mathrm{opt}}}$, ${\bar{W}}_{{\bar{E}}_{\mathrm{opt}}}$, $\eta_{{\bar{E}}_{\mathrm{opt}}}$, ${\bar{P}}_{{\bar{E}}_{\mathrm{opt}}}$ and ${\bar{E}}_{\mathrm{opt}}$ increase first and then decrease as π increases. As π increases, ${\left(\pi_{t}\right)}_{{\bar{W}}_{\mathrm{opt}}}$, ${\left(\pi_{t}\right)}_{{\bar{P}}_{\mathrm{opt}}}$ and ${\left(\pi_{t}\right)}_{{\bar{E}}_{\mathrm{opt}}}$ reduce first and then increase, and ${\left(\pi_{t}\right)}_{{\bar{W}}_{\mathrm{opt}}}$, ${\left(\pi_{t}\right)}_{{\bar{E}}_{\mathrm{opt}}}$, ${\left(\pi_{t}\right)}_{\eta_{\mathrm{opt}}}$ and ${\left(\pi_{t}\right)}_{{\bar{P}}_{\mathrm{opt}}}$ reached the minimum successively. As π increases, ${\left(v_{5}/v_{1}\right)}_{{\bar{W}}_{\mathrm{opt}}}$, and ${\left(v_{5}/v_{1}\right)}_{{\bar{E}}_{\mathrm{opt}}}$ decline, and their values have little difference. ${\left(u_{H}\right)}_{{\bar{W}}_{\mathrm{opt}}}$, ${\left(u_{H}\right)}_{\eta_{\mathrm{opt}}}$, ${\left(u_{H}\right)}_{{\bar{P}}_{\mathrm{opt}}}$ and ${\left(u_{H}\right)}_{{\bar{E}}_{\mathrm{opt}}}$ decrease as π increases, and ${\left(u_{H}\right)}_{\eta_{\mathrm{opt}}}$ is always the smallest. ${\left(u_{H1}\right)}_{{\bar{W}}_{\mathrm{opt}}}$ and ${\left(u_{H1}\right)}_{{\bar{E}}_{\mathrm{opt}}}$ rise firstly and then tend to keep constant as π rises. ${\left(u_{H1}\right)}_{{\bar{P}}_{\mathrm{opt}}}$ first increases then decreases and finally tends to stay stable as π rises. ${\left(u_{L}\right)}_{{\bar{W}}_{\mathrm{opt}}}$, ${\left(u_{L}\right)}_{{\bar{P}}_{\mathrm{opt}}}$ and ${\left(u_{L}\right)}_{{\bar{E}}_{\mathrm{opt}}}$ first increase rapidly and then slowly as π increases.

#### 3.2.2. Influences of Temperature Ratios on Optimization Results

**Figure 13:**
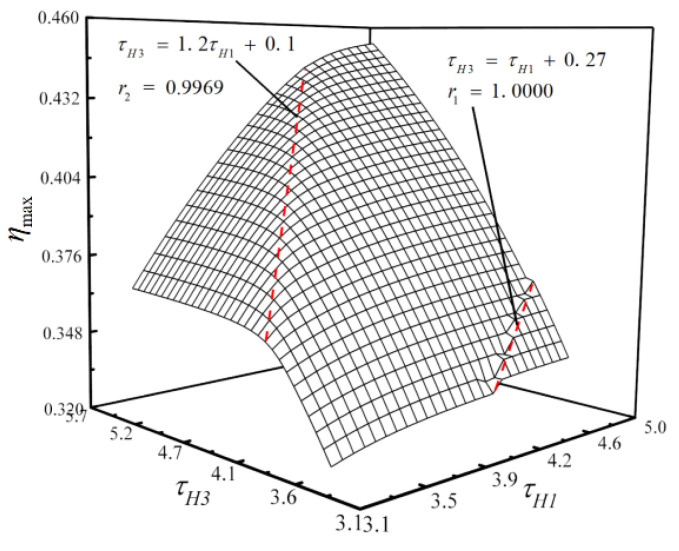
Relationships of $\eta_{\max}$ versus $\tau_{H1}$ and $\tau_{H3}$.

With η as the performance indicator, the influences of the temperature ratios on the optimization results are discussed. The relationship of the maximum thermal efficiency ($\eta_{\max}$) versus $\tau_{H1}$ and $\tau_{H3}$ is shown in Figure 13. According to Figure 12, the surface is divided into three parts by line $\tau_{H3}=\tau_{H1}+0.27$ (the correlation coefficient is $r_{1}=0.9969$) and $\tau_{H3}=1.2\tau_{H1}+0.1$ (the correlation coefficient is $r_{2}=1.0000$). $\tau_{H1}$ has little influence on $\eta_{\max}$. When $\tau_{H3}<1.2\tau_{H1}+0.1$, $\eta_{\max}$ increases as $\tau_{H3}$ increases; when $\tau_{H3}>1.2\tau_{H1}+0.1$, $\tau_{H3}$ has little impact on $\eta_{\max}$. It is recommended to magnify $\tau_{H1}$.

By numerical calculations, the surface is divided into three parts by line $\tau_{H3}=0.84\tau_{H1}+0.41$ (the correlation coefficient is $r_{1}=0.9973$) and $\tau_{H3}=1.2\tau_{H1}+0.23$ (the correlation coefficient is $r_{2}=0.9988$) with $\bar{W}$ as the performance indicator. The surface is divided into three parts by line $\tau_{H3}=0.78\tau_{H1}+0.6$ (the correlation coefficient is $r_{1}=0.9574$) and $\tau_{H3}=1.2\tau_{H1}+0.33$ (the correlation coefficient is $r_{2}=0.9991$) with $\bar{P}$ as the performance indicator. The surface is divided into three parts by line $\tau_{H3}=0.93\tau_{H1}+0.058$ (the correlation coefficient is $r_{1}=0.9978$) and $\tau_{H3}=1.1\tau_{H1}+0.41$ (the correlation coefficient is $r_{2}=0.9990$) with $\bar{E}$ as the performance indicator. In practice, the difference between $\tau_{H1}$ and $\tau_{H3}$ should be controlled and should not be too large.

#### 3.2.3. Influences of the Compressor and the Turbine’s Irreversibilities on Optimization Results

**Figure 14:**
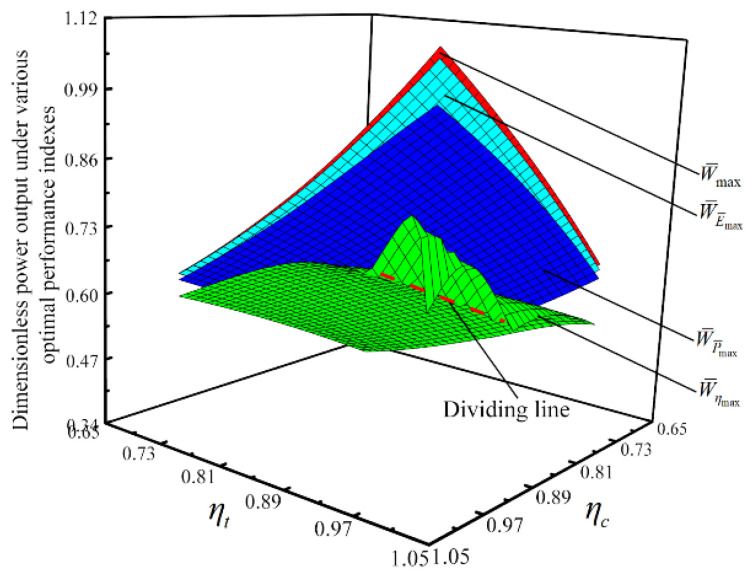
Relationships of $\bar{W}$ under various optimal performance indexes versus $\eta_{c}$ and $\eta_{t}$.

**Figure 15:**
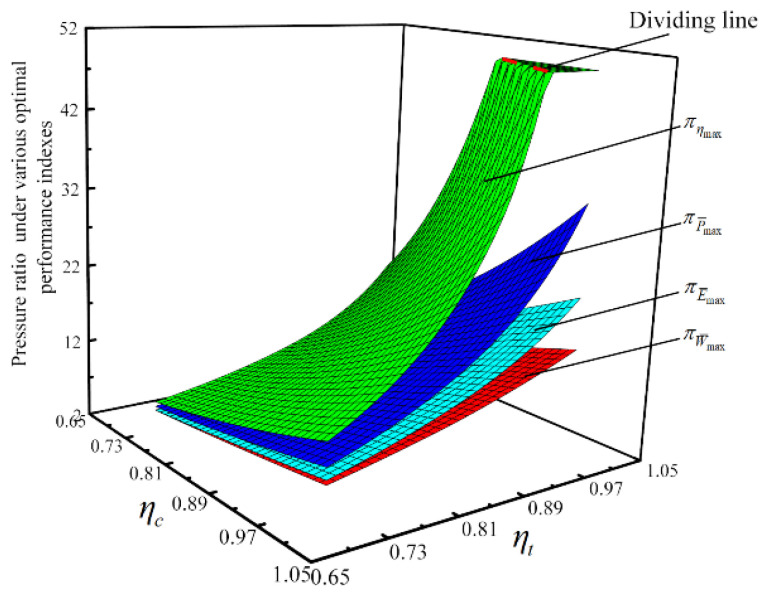
Relationships of π under various optimal performance indexes versus $\eta_{c}$ and $\eta_{t}$.

With the four performance indicators as OPOs, respectively, the influences of $\eta_{c}$ and $\eta_{t}$ on optimization results are considered, and the thermodynamic parameters under various optimal performance indicators are compared. Figure 14 and Figure 15 show relationships of $\bar{W}$ and π under various optimal performance indicators versus $\eta_{c}$ and $\eta_{t}$, respectively ${\bar{W}}_{\max}$, ${\bar{P}}_{\max}$, and ${\bar{E}}_{\max}$ are the maximum dimensionless power output, maximum dimensionless power density, and maximum dimensionless ecological function, respectively. When ${\bar{W}}_{\max}$, $\eta_{\max}$, ${\bar{P}}_{\max}$, and ${\bar{E}}_{\max}$ are used as subscripts, they indicate the corresponding values at ${\bar{W}}_{\max}$, $\eta_{\max}$, ${\bar{P}}_{\max}$, and ${\bar{E}}_{\max}$ points.

As shown in Figure 14, $\bar{W}$ under various optimal performance indicators increases as $\eta_{c}$ or $\eta_{t}$ increases. When $\eta_{c}$ and $\eta_{t}$ both approach 1, ${\bar{W}}_{\eta_{\max}}$ first increases and then decreases as $\eta_{c}$ or $\eta_{t}$ increases. When $\eta_{c}=\eta_{t}=1$, η rises monotonically as π gains, and there is no maximum value. In the case of the same $\eta_{c}$ and $\eta_{t}$, there is ${\bar{W}}_{\max}>{\bar{W}}_{{\bar{E}}_{\max}}>{\bar{W}}_{{\bar{P}}_{\max}}>{\bar{W}}_{\eta_{\max}}$. As shown in Figure 15, π under various optimal performance indicators all increase as $\eta_{c}$ or $\eta_{t}$ increases. But the influence of $\eta_{t}$ on π is more significant than that of $\eta_{c}$ on π. When $\eta_{c}$ and $\eta_{t}$ both approach 1, $\pi_{\eta_{\max}}$ is always 50. Because the upper limit of π is 50. In the case of the same $\eta_{c}$ and $\eta_{t}$, there is $\pi_{\eta_{\max}}>\pi_{{\bar{P}}_{\max}}>\pi_{{\bar{E}}_{\max}}>\pi_{{\bar{W}}_{\max}}$. The given range of π is 2≤π≤50, so when π=50, the trends of ${\bar{W}}_{\eta_{\max}}$ and $\pi_{\eta_{\max}}$ change significantly.

By numerical calculations, η, $\bar{P}$, and $\bar{E}$ under various optimal performance indicators increases as $\eta_{c}$ or $\eta_{t}$ increases. When $\eta_{c}$ and $\eta_{t}$ both approach 1, ${\bar{P}}_{\eta_{\max}}$ and ${\bar{E}}_{\eta_{\max}}$ first rises and then drops as $\eta_{c}$ or $\eta_{t}$ rises. In the same $\eta_{c}$ and $\eta_{t}$, there are $\eta_{\max}>\eta_{{\bar{P}}_{\max}}>\eta_{{\bar{E}}_{\max}}>\eta_{{\bar{W}}_{\max}}$, ${\bar{P}}_{\max}>{\bar{P}}_{{\bar{E}}_{\max}}>{\bar{P}}_{{\bar{W}}_{\max}}>{\bar{P}}_{\eta_{\max}}$, (when $\eta_{c}$ and $\eta_{t}$ both tend to 1, the relationship does not work) and ${\bar{E}}_{\max}>{\bar{E}}_{{\bar{P}}_{\max}}>{\bar{E}}_{{\bar{W}}_{\max}}>{\bar{E}}_{\eta_{\max}}$ (the difference between ${\bar{E}}_{{\bar{P}}_{\max}}$ and ${\bar{E}}_{{\bar{W}}_{\max}}$ is very small).

The calculations also show that the thermal capacitance rate matchings among the VTHRs and working fluid have influences on the cycle performance. ${\bar{W}}_{\max}$, $\eta_{\max}$, ${\bar{P}}_{\max}$, and ${\bar{E}}_{\max}$ increase first and then keep constants as $C_{H}/C_{wf}$ or $C_{H1}/C_{wf}$ increases, and the effects of $C_{H}/C_{wf}$ on ${\bar{W}}_{\max}$, $\eta_{\max}$, ${\bar{P}}_{\max}$, and ${\bar{E}}_{\max}$ are more significant than that of $C_{H1}/C_{wf}$.

## 4. Multi-Objective Optimization

### 4.1. Optimization Algorithm and Decision-Making Methods

**Figure 16:**
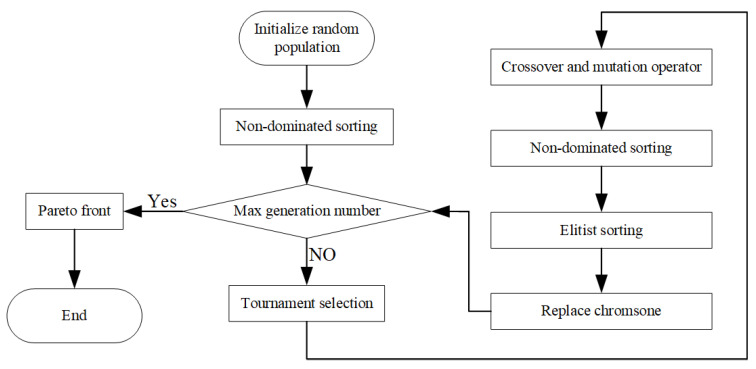
Flowchart of NSGA-II algorithm.

It is impossible to achieve the maximums of $\bar{W}$, η, $\bar{P}$, and $\bar{E}$ under the same π. It shows that there is a contradiction among the four performance indicators. The multi-objective optimization problem is solved by applying the NSGA-II algorithm [99,100,102,105,106,107,108,109,110,111,112,113,114,115,116,117,118,119,120,121,122,123,124,125]. The detailed optimization process is shown in Figure 16. The Pareto frontier of the cycle performance is obtained by taking $\bar{W}$, η, $\bar{P}$, and $\bar{E}$ as OPOs, using the NSGA-II algorithm. The optimal scheme is selected by using the LINMAP, TOPSIS, and Shannon Entropy methods [99,102], and the algorithm of “gamultiobj” in MATLAB is based on the NSGA-II algorithm. The calculations are assisted by applying the “gamultiobj”, and the corresponding Pareto frontier could be obtained. The parameter settings of “gamultiobj” are listed in Table 1.

The positive and negative ideal points are the optimal and inferior schemes of each performance indicator. The LINMAP method is the Euclidian distance between each scheme and the positive ideal point, among which the one with the smallest distance is the best scheme. Suppose that the Pareto front contains *n* feasible solutions, and each viable solution contains *m* objective values $F_{ij}$(1≤i≤m and 1≤j≤n). After normalizing $F_{ij}$, the value $B_{ij}$ is:

(79) $$B_{ij}=F_{ij}/\sqrt{\sum_{i=1}^{n}F_{ij}^{2}}$$

The weight of the *j*-th OPO is $w_{j}^{\mathrm{LINMAP}}$, and the weighted value of $B_{ij}$ is $G_{ij}$:

(80) $$G_{ij}=w_{j}^{\mathrm{LINMAP}}\cdot B_{ij}\;$$

The *j*-th objective of the positive ideal point is normalized and weighted, and the corresponding value is $G_{j}^{\mathrm{positive}}$. The Euclidean distance between the *i*-th feasible solution and the positive ideal point is $ED_{i}^{+}$:

(81) $$ED_{i}^{+}=\sqrt{\sum_{j=1}^{m}{\left(G_{ij}-G_{j}^{\mathrm{positive}}\right)}^{2}}$$

The best viable solution to the LINMAP method is $i_{\mathrm{opt}}$:

(82) $$i_{\mathrm{opt}}\in \min\{ED_{i}^{+}\}$$

The TOPSIS method considers the Euclidean distance among each scheme and the positive and negative ideal points comprehensively, to further obtain the best scheme. The weight of the *j*-th OPO is $w_{j}^{\mathrm{TOPSIS}}$, and the weighted value of $B_{ij}$ is $G_{ij}$:

(83) $$G_{ij}=w_{j}^{\mathrm{TOPSIS}}\cdot B_{ij}$$

The *j*-th objective of the negative ideal point is normalized and weighted, and the corresponding value is $G_{j}^{\mathrm{negative}}$. The Euclidean distance between the *i*-th feasible solution and the negative ideal point is $ED_{i}^{-}$:

(84) $$ED_{i}^{-}=\sqrt{\sum_{j=1}^{m}{\left(G_{ij}-G_{j}^{\mathrm{negative}}\right)}^{2}}$$

The best feasible solution of the TOPSIS method is $i_{\mathrm{opt}}$:

(85) $$i_{\mathrm{opt}}\in \min\{\frac{ED_{i}^{-}}{ED_{i}^{+}+ED_{i}^{-}}\}$$

The Shannon Entropy method is a method to get the weight of multi-attribute decision-making.

After normalization of $F_{ij}$, $P_{ij}$ is obtained:

(86) $$P_{ij}=F_{ij}/\sum_{i=1}^{n}F_{ij}$$

The Shannon Entropy and weight of the *j*-th OPO are:

(87) $$SE_{j}=-\frac{1}{\ln n}\sum_{i=1}^{n}P_{ij}\ln P_{ij}$$

(88) $$w_{j}^{\mathrm{Shannon}\;\mathrm{Entropy}}=(1-SE_{j})/\sum_{j=1}^{n}\left(1-SE_{j}\right)$$

The best feasible solution of the TOPSIS method is $i_{\mathrm{opt}}$:

(89) $$i_{\mathrm{opt}}\in \min\{P_{ij}\cdot w_{j}^{\mathrm{Shannon}\;\mathrm{Entropy}}\}$$

The deviation index *D* is defined as:

(90) $$D=\frac{\sqrt{\sum_{j=1}^{m}{\left(G_{i_{\mathrm{opt}}j}-G_{j}^{\mathrm{positive}}\right)}^{2}}}{\sqrt{\sum_{j=1}^{m}{\left(G_{i_{\mathrm{opt}}j}-G_{j}^{\mathrm{positive}}\right)}^{2}}+\sqrt{\sum_{j=1}^{m}{\left(G_{i_{\mathrm{opt}}j}-G_{j}^{\mathrm{positive}}\right)}^{2}}}$$

In this paper, $w_{j}^{\mathrm{LINMAP}}=w_{j}^{\mathrm{TOPSIS}}=1$ is chosen for the convenience of calculation.

### 4.2. Multi-Objective Optimization Results

**Figure 17:**
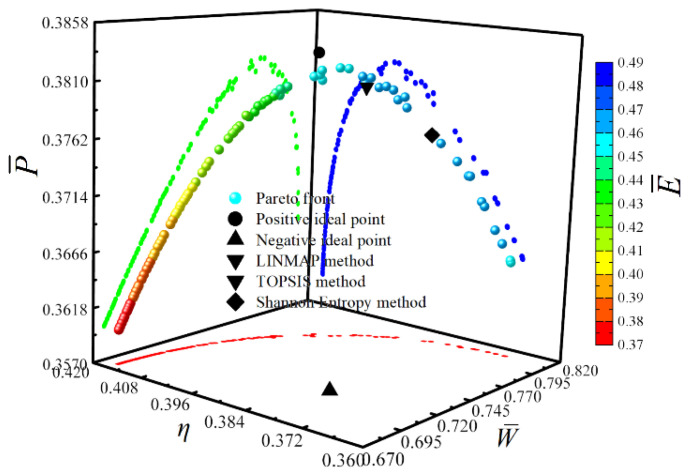
Pareto frontier and optimal schemes corresponding to the four objectives ($\bar{W}$, η, $\bar{P}$ and $\bar{E}$ ) optimization.

**Figure 18:**
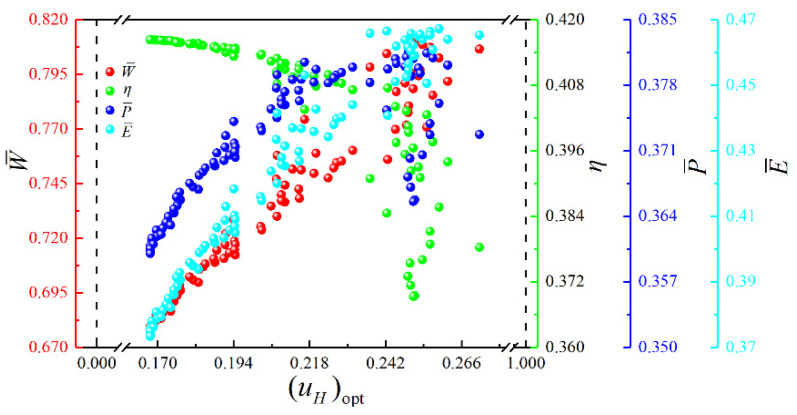
Distribution of ${\left(u_{H}\right)}_{\mathrm{opt}}$ within the value range in the Pareto frontier.

**Figure 19:**
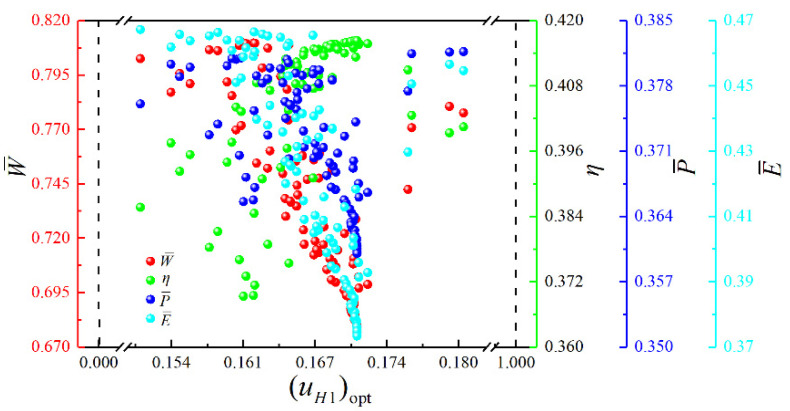
Distribution of ${\left(u_{H1}\right)}_{\mathrm{opt}}$ within the value range in the Pareto frontier.

**Figure 20:**
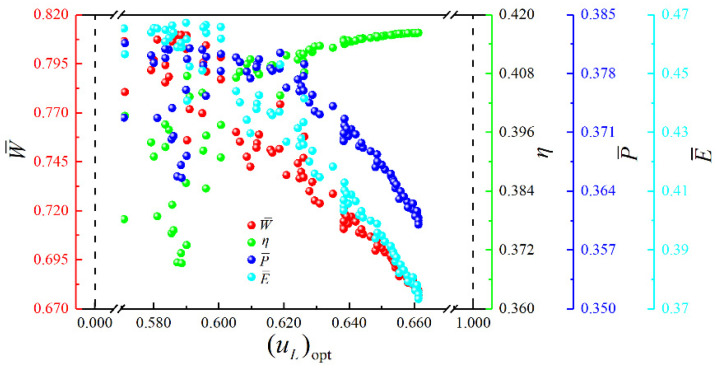
Distribution of ${\left(u_{L}\right)}_{\mathrm{opt}}$ within the value range in the Pareto frontier.

**Figure 21:**
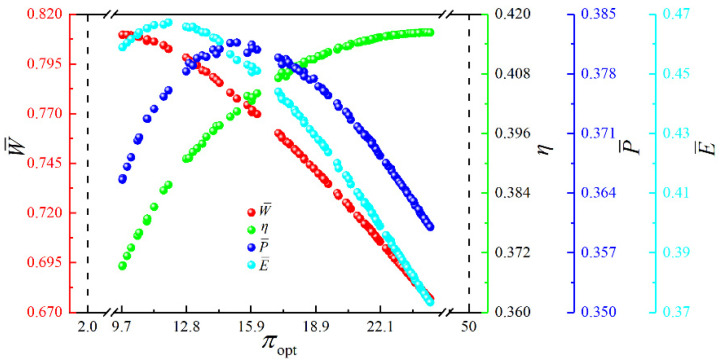
Distribution of $\pi_{\mathrm{opt}}$ within the value range in the Pareto frontier.

Figure 17 shows the Pareto frontier and optimal schemes corresponding to the four objectives ($\bar{W}$, η, $\bar{P}$ and $\bar{E}$) optimization. The color on the Pareto frontier denotes the size of $\bar{E}$. To facilitate the observation of the changing relationships among the objectives, the pure red projection indicates the changing relationship between $\bar{W}$ and η. The pure green projection shows the changing relationship between $\bar{W}$ and $\bar{P}$, and the pure blue projection indicates the changing relationship between η and $\bar{P}$. It is easy to know that $\bar{W}$ and η, $\bar{W}$ and $\bar{P}$, η and $\bar{P}$ are all parabolic-like relationships with the opening downward. To analyze the influence of the corresponding optimization variables (${\left(u_{H}\right)}_{\mathrm{opt}}$, ${\left(u_{H1}\right)}_{\mathrm{opt}}$, ${\left(u_{L}\right)}_{\mathrm{opt}}$ and $\pi_{\mathrm{opt}}$) on cycle performance, the distributions of ${\left(u_{H}\right)}_{\mathrm{opt}}$, ${\left(u_{H1}\right)}_{\mathrm{opt}}$, ${\left(u_{L}\right)}_{\mathrm{opt}}$ and $\pi_{\mathrm{opt}}$ within the Pareto frontier’s value range are shown in Figure 18, Figure 19, Figure 20 and Figure 21. As shown in Figure 18, the value range of ${\left(u_{H}\right)}_{\mathrm{opt}}$ is 0–1, but its distribution is between 0.167 and 0.272. As ${\left(u_{H}\right)}_{\mathrm{opt}}$ increases, $\bar{W}$, $\bar{P}$, and $\bar{E}$ gradually increase, but η gradually decreases. As shown in Figure 19, the value range of ${\left(u_{H1}\right)}_{\mathrm{opt}}$ is 0–1, but its distribution is between 0.151 and 0.181. As ${\left(u_{H1}\right)}_{\mathrm{opt}}$ increases, $\bar{W}$, $\bar{P}$, and $\bar{E}$ gradually decrease, but the changing trend of η is not apparent. As shown in Figure 20, the value range of ${\left(u_{L}\right)}_{\mathrm{opt}}$ is 0–1, but its distribution is between 0.568 and 0.662. As ${\left(u_{L}\right)}_{\mathrm{opt}}$ increases, $\bar{W}$, $\bar{P}$, and $\bar{E}$ gradually decrease, but the changing trend of η is not apparent. As shown in Figure 21, the value range of $\pi_{\mathrm{opt}}$ is 2–50, but its distribution is between 9.692 and 24.426. As $\pi_{\mathrm{opt}}$ increases, $\bar{W}$ gradually decreases, η gradually increases, and $\bar{P}$ and $\bar{E}$ rise and then reduce.

The Pareto frontier includes a series of non-inferior solutions, so the appropriate solution must be chosen according to the actual situation. The results of the triple- and double-objective optimizations are further discussed to compare the results of multi-objective optimizations more comprehensively. The comparison of the optimal schemes gotten by single- and double-, triple-, and quadruple-objective optimizations are listed in Table 2. The deviation index (*D*) is applied to represent the proximity between the optimal scheme and the positive ideal point. The appropriate optimal schemes are chosen by using the three methods. For the quadruple-objective optimization, $\bar{W}$, η, $\bar{P}$, and $\bar{E}$ corresponding to the positive ideal point are the maximum of the single-objective optimization. It indicates that the Pareto frontier includes all single-objective optimization results. The *D* obtained by the Shannon Entropy method is significantly smaller than that obtained by the LINMAP and TOPSIS methods. Simultaneously, it can be found that the *D* obtained by the Shannon Entropy method is the same as that with $\bar{E}$ as the OPO. For the triple-objective optimization, the triple-objective ($\bar{W}$, η and $\bar{E}$) optimization *D* obtained by the LINMAP or TOPSIS method is the smallest. For the double-objective optimization, the double-objective ($\bar{W}$ and $\bar{P}$) optimization *D* obtained by the LINMAP method is the smallest. For the single-objective optimization, the *D* corresponding to ${\bar{E}}_{\max}$ is the smallest. For single- and double-, triple-, and quadruple-objective optimizations, the double-objective ($\bar{W}$ and $\bar{P}$) optimization *D* obtained by the LINMAP method is the smallest.

## 5. Conclusions

Based on FTT, an improved irreversible closed modified simple BCY model with one IHP and coupled to VTHRs is established and optimized with four performance indicators as OPOs, respectively. The optimization results are compared, and the influences of compressor and turbine efficiencies on optimization results are analyzed. Finally, the cycle is optimized, and the corresponding Pareto frontier is gained by adopting the NSGA-II algorithm. Based on three different methods, the optimal scheme is gotten from the Pareto frontier. The results obtained in this paper reveal the original results in Refs. [10,11,12], which were the initial work of the FTT theory. The main results are summarized:

1. For the single-objective analyses and optimizations, performance indicators all rise as $\eta_{c}$ and $\eta_{t}$ rise. The influences of $\eta_{t}$ on four performance indicators are greater than those of $\eta_{c}$. $\bar{W}$ of the models in this paper increase and then decrease as π increases in both cases; that is, the qualitative law is the same. However, there is an apparent quantitative difference between the two points. In practice, the difference between $\tau_{H1}$ and $\tau_{H3}$ should be controlled and not be too large. $\bar{P}$ and $\bar{E}$ are the trade-offs between $\bar{W}$ and η.
2. For single- and double-, triple-, and quadruple-objective optimizations, the Pareto frontier includes a series of non-inferior solutions. The appropriate solution could be chosen according to the actual situation. By comparison, it is found that the double-objective ($\bar{W}$ and $\bar{P}$) optimization *D* obtained by the LINMAP method is the smallest.
3. The optimization results gained in this paper could offer theoretical guidelines for the optimal designs of the gas turbine plants. In the next step, the improved closed intercooling regenerated modified BCY model with one IHP will be optimized with real gas as the working fluid, and the internal friction-based pressure drops during heating and cooling processes and other processes, as well as the heat leakage losses between the heat source and the environment, will be taken into account.

## Figures and Tables

**Table 1:** Parameter settings of “gamultiobj”.

Parameters	Values
Nvars	4
ParetoFraction	0.3
PopulationSize	300
Generations	500
CrossoverFraction	0.8

**Table 2:** Comparison of the optimal schemes gotten by the single- and double-, triple-, and quadruple-objective optimizations.

OPOs	Decision Methods	Optimization Variables				Performance Indicators				Isothermal Pressure Drop Ratio	Deviation Indexes
		$u_{H}$	$u_{H1}$	$u_{L}$	π	$\bar{W}$	η	$\bar{P}$	$\bar{E}$	$\pi_{t}$	D
$\bar{W}$, η, $\bar{P}$, and $\bar{E}$	LINMAP	0.245	0.154	0.601	14.194	0.787	0.397	0.380	0.462	0.572	0.172
	TOPSIS	0.245	0.154	0.601	14.194	0.787	0.397	0.380	0.462	0.572	0.172
	Shannon Entropy	0.259	0.151	0.590	11.901	0.802	0.386	0.376	0.467	0.572	0.167
$\bar{W}$, η, and $\bar{P}$	LINMAP	0.230	0.167	0.603	14.261	0.787	0.398	0.381	0.461	0.557	0.170
	TOPSIS	0.231	0.167	0.602	14.115	0.788	0.397	0.380	0.462	0.557	0.168
	Shannon Entropy	0.246	0.167	0.587	12.008	0.803	0.386	0.377	0.466	0.559	0.165
$\bar{W}$, η, and $\bar{E}$	LINMAP	0.231	0.163	0.606	13.947	0.790	0.397	0.380	0.462	0.557	0.160
	TOPSIS	0.231	0.163	0.606	13.947	0.790	0.397	0.380	0.462	0.557	0.160
	Shannon Entropy	0.257	0.153	0.590	11.92	0.803	0.386	0.376	0.467	0.570	0.165
$\bar{W}$, $\bar{P}$ and $\bar{E}$	LINMAP	0.252	0.177	0.571	13.339	0.793	0.393	0.380	0.463	0.566	0.162
	TOPSIS	0.252	0.177	0.571	13.339	0.793	0.393	0.380	0.463	0.566	0.162
	Shannon Entropy	0.259	0.151	0.590	11.906	0.802	0.386	0.376	0.467	0.572	0.167
η, $\bar{P}$ and $\bar{E}$	LINMAP	0.241	0.170	0.589	17.016	0.761	0.406	0.380	0.444	0.575	0.319
	TOPSIS	0.241	0.170	0.589	17.016	0.761	0.406	0.380	0.444	0.575	0.319
	Shannon Entropy	0.245	0.169	0.585	16.674	0.764	0.405	0.381	0.447	0.577	0.297
$\bar{W}$ and η	LINMAP	0.230	0.169	0.601	14.293	0.787	0.398	0.381	0.461	0.557	0.170
	TOPSIS	0.230	0.169	0.601	14.293	0.787	0.398	0.381	0.461	0.557	0.170
	Shannon Entropy	0.248	0.168	0.585	12.061	0.802	0.387	0.377	0.466	0.560	0.162
$\bar{W}$ and $\bar{P}$	LINMAP	0.247	0.176	0.578	13.384	0.793	0.394	0.380	0.463	0.563	0.158
	TOPSIS	0.247	0.176	0.577	13.560	0.792	0.394	0.381	0.463	0.564	0.161
	Shannon Entropy	0.245	0.171	0.584	11.855	0.803	0.385	0.376	0.466	0.555	0.170
$\bar{W}$ and $\bar{E}$	LINMAP	0.258	0.154	0.589	11.765	0.803	0.385	0.376	0.467	0.570	0.169
	TOPSIS	0.258	0.154	0.589	11.765	0.803	0.385	0.376	0.467	0.570	0.169
	Shannon Entropy	0.259	0.152	0.589	11.902	0.802	0.386	0.376	0.467	0.572	0.167
η and $\bar{P}$	LINMAP	0.232	0.192	0.576	16.452	0.765	0.405	0.381	0.446	0.562	0.295
	TOPSIS	0.235	0.193	0.572	16.156	0.768	0.404	0.381	0.447	0.563	0.279
	Shannon Entropy	0.241	0.196	0.563	15.603	0.772	0.402	0.381	0.450	0.564	0.255
η and $\bar{E}$	LINMAP	0.237	0.1604	0.603	14.307	0.787	0.398	0.381	0.461	0.564	0.170
	TOPSIS	0.236	0.163	0.601	14.173	0.788	0.398	0.381	0.462	0.562	0.164
	Shannon Entropy	0.258	0.152	0.590	11.909	0.802	0.386	0.376	0.467	0.571	0.167
$\bar{P}$ and $\bar{E}$	LINMAP	0.257	0.166	0.578	13.483	0.792	0.394	0.380	0.464	0.572	0.160
	TOPSIS	0.257	0.165	0.578	13.386	0.793	0.393	0.380	0.464	0.572	0.161
	Shannon Entropy	0.258	0.154	0.588	12.054	0.802	0.387	0.377	0.467	0.571	0.161
$\bar{W}$		0.249	0.162	0.589	9.678	0.810	0.369	0.365	0.459	0.550	0.242
η		0.152	0.174	0.674	24.542	0.672	0.416	0.358	0.369	0.532	0.783
$\bar{P}$		0.251	0.183	0.567	15.149	0.777	0.400	0.382	0.454	0.571	0.225
$\bar{E}$		0.259	0.151	0.590	11.903	0.802	0.386	0.376	0.467	0.572	0.167
Positive ideal point		——	——	——	——	0.810	0.416	0.382	0.467	0.810	——
Negative ideal point		——	——	——	——	0.677	0.369	0.360	0.373	0.677	——

## Nomenclature

a, x, y	Intermediate variables
C	Thermal capacity rate (kW/K)
$C_{p}$	Specific heat at constant pressure (kJ/(kg·K))
E	Effectiveness of heat exchanger or ecological function (kW)
$\bar{E}$	Dimensionless ecological function
k	Specific heat ratio
M	Mach number
N	Number of the heat transfer unit
$\dot{Q}$	Heat absorbing rate or heat releasing rate (kW)
$\bar{P}$	Dimensionless power density
T	Temperature (K)
U	Heat conductance (kW/K)
u	Heat conductance distribution
$\bar{W}$	Dimensionless power output
**Greek symbols**	
η	Efficiency
π	Pressure ratio
τ	Temperature ratio
**Subscripts**	
H	Hot-side heat exchanger
L	Cold-side heat exchanger
wf	Working fluid
1,2,3,4,5,2s,5s	State points

## Abbreviations

Brayton cycle	BCY
CCC	Convergent combustion chamber
CTHR	Constant-temperature heat reservoir
FPDT	Finite Physical Dimensions Thermodynamics
FTT	Finite time thermodynamics
HCD	Heat conductance distribution
IHP	Isothermal heating process
OPO	Optimization objective
RCC	Regular combustion chamber
VTHR	Variable-temperature heat reservoir
